# SchoolHEAT: Racial and Ethnic Inequity in School Temperature

**DOI:** 10.1007/s11524-024-00919-y

**Published:** 2024-09-24

**Authors:** Kelly K. Jones, Varsha Vijay, Shannon N. Zenk

**Affiliations:** 1 Division of Intramural Research, National Institute On Minority Health and Health Disparities, 6707 Democracy Blvd, Suite 800, Bethesda, MD 20892 USA; 2 Science Based Targets Network, New York City, NY USA; 3 National Institute of Nursing Research, Bethesda, USA

**Keywords:** Land surface temperature disparities, School wellness, Climate, Heat exposure

## Abstract

Exposure to high environmental temperature is detrimental to health through multiple pathways. This paper describes disparities in school-based high-temperature exposure at metropolitan schools in the United States. Using school location and sociodemographic data from the National Center for Education Statistics, neighborhood data from the US Census Bureau, and land surface temperature (LST) data from the Aqua Earth-observing satellite mission, we find that for every 10% more Black or Hispanic residents in the neighborhood, schools have LST 0.25 °C and 0.38 °C hotter, respectively. When the Black or Hispanic student population is greater than the neighborhood population, LST is an additional 0.20 °C and 0.40 °C for each 10% increase in students over neighborhood population, respectively. Black and Hispanic students are overrepresented in the hottest schools, making up 58.7% of students in the hottest 20% of schools, compared to only 30.0% of students in the coolest 20% of schools.

**Supplementary Information:**

The online version contains supplementary material available at 10.1007/s11524-024-00919-y.

## Introduction

Climate change-induced high temperatures may increase children’s school-based exposure to heat with the potential to adversely impact health behaviors and learning outcomes. Huang et al. [1] found a significant negative association between temperature and children aged 0 to 17 participating in moderate-to-vigorous physical activity in a park setting, during which high temperatures additionally put children at increased risk of heat illness [1, 2]. Wallenberg et al. [3] similarly found decreases in physical activity among preschoolers in warm compared to cool weather in Sweden [3]. Heat also negatively impacts learning outcomes, reducing both cognitive function and standardized test scores [4, 5]. Researchers exploiting standardized school assessments have demonstrated that scores are diminished for United States (US) students by 0.04% of a standard deviation for each day hotter than 26.7 °C, and that over the entire year, a 1.5 °C increase in average temperature is associated with a 10.5% reduction in scores [6, 7]. Educational attainment is a strong predictor of adult health outcomes, with pathways ranging from increased health knowledge to greater potential adult income [8]. Thus, heat exposure has both short and long-term health implications.

Neighborhoods with higher concentrations of Black and Hispanic or low-income residents tend to be hotter than Whiter and higher income neighborhoods, both in the US and around the world [9–11]. Dialesandro et al. [9] and Hsu et al. [10] found that in US metropolitan areas, non-White and high poverty neighborhoods are expected to be between 0.5 and 4 °C warmer than majority White or wealthy neighborhoods, while Chakraborty et al. [11] showed that in half of the 25 global cities they examined, the urban heat island intensity was distributed such that the heat burden fell disproportionately on low-income residents. Several recent US studies have shown that historical redlining in a neighborhood is associated with higher contemporary temperatures [12–15].

While much work has been done examining heat distribution across neighborhoods and within schoolyards (e.g., playground areas), we are unaware of any national analysis that has looked at temperatures at school sites broadly, including the totality of the school surrounds (e.g., schoolyard, parking areas, surrounding walkways and/or green spaces), or considered the combination of neighborhood and school sociodemographics [16–19]. Schools are of particular importance because they are special resources in communities, both for the students and the neighboring residents [20–22]. School conditions may be uniquely amenable to policy interventions because funding mechanisms are already in place to make changes to the built environment or initiate supportive programming.

Climate scientists have concluded that for much of the US, and especially urban areas, summertime heating will become more extreme, including the continuation of high temperatures later into the Fall, when children are in school [23]. Increased risk accompanies these changes in heat conditions [24]. Given that conditions will continue to worsen, it is imperative to understand what disparities exist currently, in order to adequately prepare for the future.

In this study, we use a national dataset of schools, including precise locations and school catchment areas, and satellite-derived outdoor temperatures to investigate disparities in temperatures at public school locations in metropolitan and micropolitan areas of the contiguous US. Black, Hispanic, and White students make up nearly 90% of the school population; the other students are defined as American Indian and Alaska Native (0.7% of total students), Asian American and Pacific Islanders (5.5% of total students), Native Hawaiian and other Pacific Islanders (0.3% of total students), and multi-racial (4.6% of total students). Insufficient variability in both school and neighborhood demographics for the lesser represented groups led us to focus this work on Black, Hispanic, and White students. Similarly, only 7.5% of students attend schools not located in metropolitan or micropolitan areas and temperatures in rural areas are less dependent on modifiable built environment factors, so we do not include those schools. We hypothesize that schools in Black and Hispanic neighborhoods, as well as schools with more Black or Hispanic students, will have higher temperatures.

## Methods

### School Data

Public school data were collected from the National Center for Education Statistics (NCES) Education Demographic and Geographic Estimates (EDGE) program [25]. We collected locations of all public schools as latitude and longitude reported through the Common Core of Data (CCD) program, and school catchment areas as polygon shapefiles through the School Area Boundary Survey (SABS) program. School catchment areas are the geographic areas in which resident children are assigned to attend a specific school and represent neighborhoods in this project. The most recent SABS data are from school year 2015–2016; we assume temporal stability in catchment areas. We collected public school demographics from the CCD using the Elementary/Secondary Information System (ElSi) data tool for school year 2020–2021 [26]. For each school, we collected the grade levels served, total students, and race/ethnicity of students. Schools were classified as elementary schools if they had students in grade 5 or below, as middle schools if they had students in grades 6, 7, or 8, and as high schools if they had students in grades 9 through 12. This resulted in some areas having multiple schools of the same classification and some schools having multiple classifications. For example, a school where students went to the same school from kindergarten through sixth grade would be classified as both an elementary and a middle school, and if that area had a different school for seventh and eighth graders, both the school with sixth graders and the school with seventh and eighth graders were classified as middle schools. We operationalized school demographics as the proportion of students that were Black, Hispanic, or White out of the total number of students that reported race/ethnicity.

Our analytic sample of schools (*n* = 54,780) excludes schools that failed to report to either the CCD or EDGE programs (*n* = unknown), schools identified as “open enrollment” which draw their student body from a broader geographic area than traditional neighborhood schools (*n* = 4,415), and schools not located in a metropolitan or micropolitan area (*n* = 8419).

### Neighborhood Data

Neighborhood data were collected from the American Community Survey 5-year estimates 2017–2021 at the block group level (census tract for poverty) [27]. Collected variables were total population, Hispanic origin by race for all ages, poverty status in the past 12 months by age, median household income, and median home value. Census areas were assigned to school catchment areas based on centroid location.

### Land Surface Temperature

The average September afternoon land surface temperature (LST) at each school was identified by averaging the four 8-day Land Surface Temperature L3 1 km Aqua products covering the month of September each year for 2019–2022. We chose to use Aqua data because the northward pass of the Aqua satellite over the continental US occurs in the midafternoon, although it does not necessarily coincide with the hottest temperature of day. We chose to focus on September because while there is variability in the academic calendar, most schools are in session in September. School LST was defined as the average of the pixel in which the school coordinate was located and all eight neighboring cells. LST is not the same as air temperature, but the two measures are highly correlated [28, 29]; LST is used frequently in urban heat studies [9–11]. Google Earth Engine was used for satellite image processing.

### Local Region

We defined the local region to be the metro- or micropolitan statistical area (MSA) in which the school is located. MSAs are US Census-defined areas representing core urban areas and associated areas with interrelated social and economic networks [30]. Including the local region as a fixed effect in the analysis controls for sociodemographic residence patterns that correspond with climate (e.g., greater Hispanic populations are found in the Southwest, where it is generally hotter than other parts of the US) and ensures that identified effects represent deviations from regionally expected September afternoon mean temperatures. Additionally, MSAs are geographic levels that represent policy-relevant units for addressing disparities.

### Analysis

Summary statistics describe the sample. We estimated separate ordinary linear regression models to identify associations between school demographics and September afternoon school LST, controlling for neighborhood sociodemographics and including a fixed effect for MSA area. We created school demographic residual variables because school demographics depend on and are correlated with neighborhood demographics (correlations between 0.92 and 0.94). Specifically, we regressed school proportion Black, Hispanic, or White on neighborhood proportion Black, Hispanic, or White. The effect associated with the school demographic residual represents the additional additive effect of the school population deviating from the neighborhood population, beyond the neighborhood effect. This effect will be non-zero and significant if there is a separate association between LST and the school population in addition to the neighborhood effect. Elementary, middle, and high schools were modeled separately.

To understand the impact of disparate school temperature conditions on children, we classed schools by heat quintiles within the local region (e.g., quintiles defined within each MSA, regardless of quintile cutoff values across MSAs). We summed student counts within quintiles and present data on the demographics within and across heat quintiles.

### Sensitivity Analyses

We tested for sensitivity to the definition of local region, neighborhood socioeconomic status, and home neighborhood. We alternately defined local region as the state and as 5-, 10-, and 25-mile buffers around the school. We alternately defined neighborhood socioeconomic status as the median household income in the catchment and the median home value in the catchment. We alternately defined home neighborhood as the census block groups closer to focal school than any other (e.g., the Voronoi neighborhood).

We tested for sensitivity associated with any geographic change in school locations and catchment boundaries between the 2015 and 2016 school year (the latest year for which NCES EDGE data was available) and the more recent census and temperature data that we used in the analyses. We did this by collecting ACS data for the 5-year estimates spanning 2012–2016 and LST data for the years 2013–2016 and repeating all analyses.

Because weather may differ depending on region, we also tested relationships separately by region. We tested each of nine US climate regions as defined by the National Centers for Environmental Information, as follows: West (California and Nevada), Northwest (Idaho, Oregon, and Washington), Southwest (Arizona, Colorado, New Mexico, and Utah), Northern Rockies and Plains (Montana, Nebraska, North Dakota, South Dakota, and Wyoming), West (Arkansas, Kansas, Louisiana, Mississippi, Oklahoma, and Texas), Upper Midwest (Iowa, Michigan, Minnesota, and Wisconsin), Ohio Valley (Illinois, Indiana, Kentucky, Missouri, Ohio, Tennessee, and West Virginia), Southeast (Alabama, Florida, Georgia, North Carolina, South Carolina, and Virginia), and Northeast (Connecticut, Delaware, Maine, Maryland, Massachusetts, New Hampshire, New Jersey, New York, Pennsylvania, Rhode Island, and Vermont) [31].

## Results

The sample contained 54,780 unique schools representing schools in all 48 contiguous states and Washington, DC. Of these, 36,332 were categorized as elementary schools, 21,385 as middle schools, and 10,191 as high schools. Table 1 shows side-by-side comparisons of the full sample and elementary, middle, and high school samples. Overall, school populations were 13.3% Black, 25.6% Hispanic, 50.6% White, and 10.54% students of other race/ethnicity, including American Indian and Alaska Native, Asian American and Pacific Islanders, Native Hawaiian and other Pacific Islanders, and multi-racial students. In comparison, the full populations in school catchment areas were 10.5% Black, 18.0% Hispanic, and 62.7% White. On average, school populations were 2.8% more Black, 7.6% more Hispanic, and 12.1% less White than the associated catchment. The mean poverty level in catchment areas was 12.4%. The average September afternoon LST across all schools was 32.3 °C, with elementary schools averaging 32.5 °C, middle schools averaging 32.8 °C, and high schools averaging 31.4 °C.

**Table 1:** Descriptive statistics for schools, temperature, school district, and MSA

	Overall	Elementary	Middle	High
Schools (*n*)	54,780	36,332	21,385	10,191
School demographics				
School-level student total (mean, SD)	598.5, 456.26	457.87, 204.3	564.43, 334.52	1031.53, 785.93
Percent Black students (mean, SD)	13.34, 20.76	13.73, 21.30	12.16, 20.48	11.53, 19.27
Percent Hispanic students (mean, SD)	25.62, 27.00	26.79, 27.64	26.66, 28.02	20.79, 24.45
Percent White students (mean, SD)	50.59, 31.58	48.59, 31.74	51.11, 32.63	58.97, 30.81
Percent other students (mean, SD)	10.54, 11.34	10.89, 11.53	10.09, 11.52	8.73, 10.58
Catchment area demographics				
Percent Black (mean, SD)	10.53, 17.06	11.01, 17.90	9.50, 16.64	8.79, 14.78
Percent Hispanic (mean, SD)	18.03, 22.46	18.93, 23.20	18.91, 23.14	14.73, 19.82
Percent White (mean, SD)	62.71, 27.99	61.16, 28.70	62.67, 28.80	68.71, 25.78
Percent living in poverty (mean, SD)	12.39, 8.31	12.59, 8.81	12.58, 8.20	12.09, 7.06
School demographics deviation				
Deviation percent Black (mean, SD)	2.82, 8.02	2.72, 8.37	2.66, 7.73	2.75, 7.24
Deviation percent Hispanic (mean, SD)	7.59, 10.00	7.86, 10.61	7.75, 10.31	6.06, 8.61
Deviation percent White (mean, SD)	− 12.13, 11.78	− 12.57, 12.23	− 11.56, 11.77	− 9.74, 10.77
Land surface temperature (°C; mean, SD)	32.29, 6.31	32.49, 6.39	32.83, 6.90	31.41, 6.17
Schools in MSA (885 MSAs; mean, SD)	61.90, 159.82	41.05, 112.15	24.22, 65.24	11.63, 23.76

*MSA* Metropolitan/Micropolitan Statistical Area, other students include American Indian and Alaska Native, Asian American and Pacific Islanders, Native Hawaiian and other Pacific Islanders, and multi-racial students

Table 2 shows results from OLS regressions separately for elementary, middle, and high schools. Demographic coefficients have been scaled so that one unit is equivalent to a 10% population change. The top line, school demographic residual, shows the effect of school population deviations from the neighborhood population, with the second line showing the effect of the neighborhood population. Elementary school results are used to discuss the associations. As shown in Table 2, each 10% increase in the share of Black students and Hispanic students in an elementary school beyond the neighborhood share is associated with an average 0.29 °C and 0.20 °C warmer temperature, respectively, controlling for neighborhood demographics. Each 10% increase in the share of Black residents and Hispanic residents in the neighborhood is associated with an average 0.25 °C and 0.29 °C warmer temperature, respectively. For example, an elementary school in a neighborhood with 30% Black residents is expected to be 0.25 °C warmer than an elementary school in a neighborhood with 20% Black residents; if the student population is 40% Black (i.e., a 10% deviation from the neighborhood population), then the school is expected to be an additional 0.29 °C warmer. In contrast, controlling for neighborhood demographics, a 10% increase in the share of White students beyond the neighborhood share is associated with an average 0.35 °C cooler temperature. Likewise, a 10% increase in the share of neighborhood White residents is associated with an average 0.42 °C cooler temperature. Findings for neighborhood poverty are likewise consistent across racial and ethnic groups, with higher temperatures found in neighborhoods with higher poverty rates.

**Table 2:** OLS Results, student/population race/ethnicity differences in September afternoon school LST

Elementary schools			
	Black	Hispanic	White
School demographic residual	0.29 (*p* < 0.001)	0.20 (*p* < 0.001)	-0.35 (*p* < 0.001)
	0.26, 0.31	0.18, 0.22	− 0.37, − 0.34
Catchment demographics	0.25 (*p* < 0.001)	0.29 (*p* < 0.001)	− 0.42 (*p* < 0.001)
	0.23, 0.26	0.28, 0.31	− 0.44, − 0.41
Catchment poverty	0.55 (*p* < 0.001)	0.57 (*p* < 0.001)	0.11 (*p* < 0.001)
	0.52, 0.58	0.54, 0.60	0.08, 0.14
Constant	27.40 (*p* < 0.001)	27.31 (*p* < 0.001)	31.68 (*p* < 0.001)
	26.23, 28.58	26.14, 28.48	30.58, 32.79
Observations	36,332	36,332	36,332
*R*^2^	0.91	0.92	0.92
Middle schools			
	Black	Hispanic	White
School demographic residual	0.30 (*p* < 0.001)	0.22 (*p* < 0.001)	− 0.34 (*p* < 0.001)
	0.26, 0.34	0.20, 0.25	− 0.36, − 0.32
Catchment demographics	0.27 (*p* < 0.001)	0.30 (*p* < 0.001)	− 0.45 (*p* < 0.001)
	0.25, 0.29	0.28, 0.32	− 0.47, − 0.43
Catchment poverty	0.56 (*p* < 0.001)	0.59 (*p* < 0.001)	0.08 (*p* < 0.001)
	0.52, 0.60	0.55, 0.63	0.04, 0.12
Constant	26.96 (*p* < 0.001)	26.96 (*p* < 0.001)	31.60 (*p* < 0.001)
	25.65, 28.28	25.65, 28.22	30.36, 32.85
Observations	21,385	21,385	21,385
*R*^2^	0.93	0.93	0.94
High schools			
	Black	Hispanic	White
School demographic residual	0.40 (*p* < 0.001)	0.23 (*p* < 0.001)	− 0.33 (*p* < 0.001)
	0.34, 0.46	0.18, 0.28	− 0.37, − 0.29
Catchment demographics	0.35 (*p* < 0.001)	0.38 (*p* < 0.001)	− 0.49 (*p* < 0.001)
	0.31, 0.38	0.34, 0.41	− 0.51, − 0.46
Catchment poverty	0.53 (*p* < 0.001)	0.57 (*p* < 0.001)	0.02 (*p* = 0.50)
	0.45, 0.60	0.50, 0.64	− 0.05, 0.10
Constant	26.85 (*p* < 0.001)	26.74 (*p* < 0.001)	31.85 (*p* < 0.001)
	25.35, 28.35	25.24, 28.24	30.40, 33.29
Observations	10,191	10,191	10,191
*R*^2^	0.92	0.92	0.92

Coefficients, *p*-values in parentheses, and 95% confidence intervals for independently run models. All models included MSA-level fixed effects

These patterns hold across school levels, with temperatures in neighborhoods with more Black or Hispanic residents, and effects associated with school population residuals of similar magnitudes, with opposite associations with White neighborhood and school populations.

**Fig. 1:**
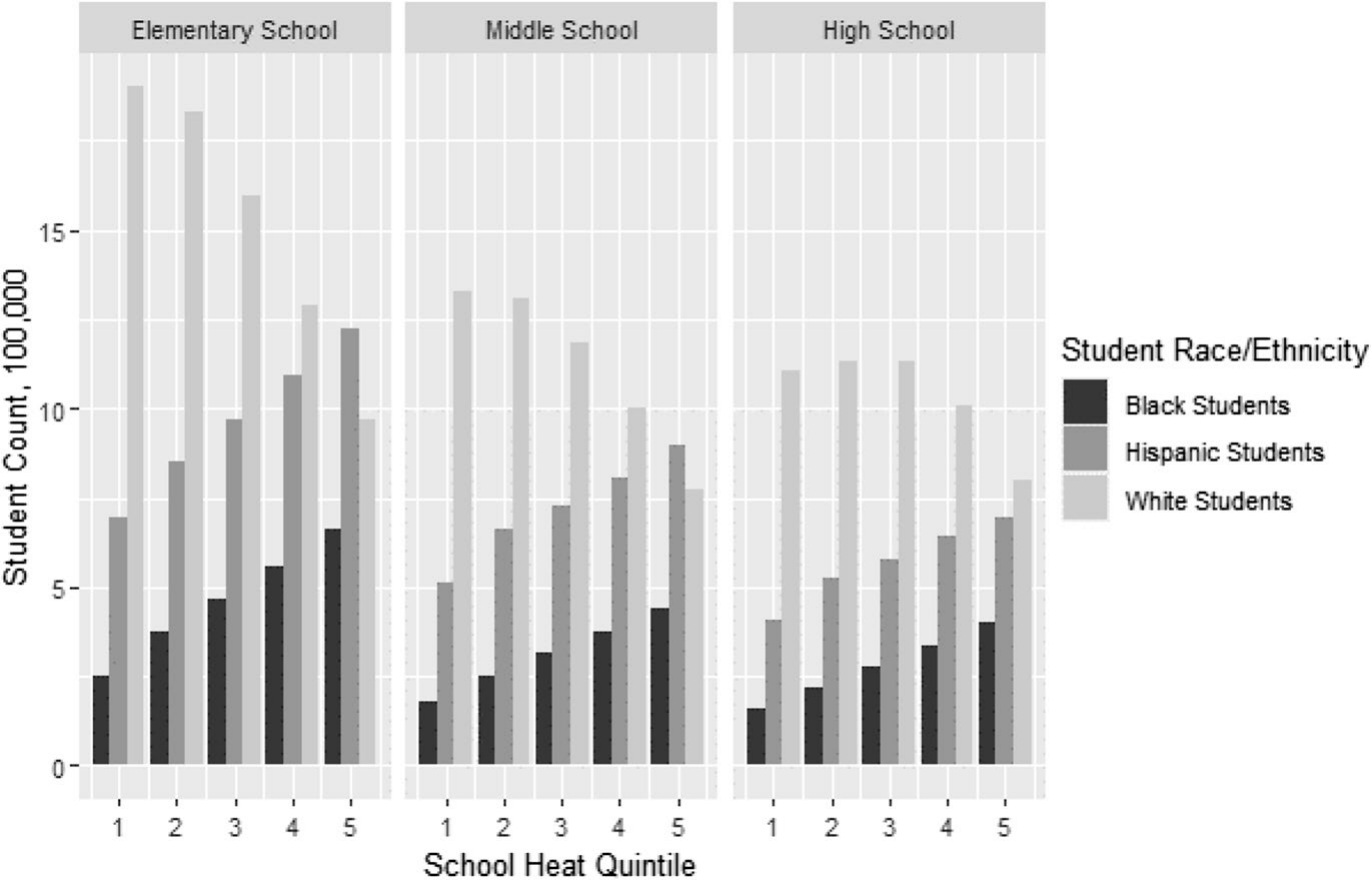
Distribution of students in school heat quintiles by race/ethnicity for elementary, middle, and high schools. Note: Heat Quintiles go from 1 (coolest) to 5 (hottest) and are classified within MSA. Bars represent number of students of each race/ethnicity in all the schools in the heat quintile, in 100,000

Figure 1 shows the distribution of Black, White, and Hispanic students across schools with different exposure to heat, categorized by school temperature quintile. Again, elementary schools are used to discuss the results. Out of 16.6 million elementary school students in our sample, 7.6 million (45.6%) are White, 4.8 million (29.1%) are Hispanic, 2.3 million (13.9%) are Black, and 1.9 million (11.5%) are of other race/ethnicity. Of the White students, 1.9 million (25.0%) are enrolled in the coolest 20% of schools, while 1.0 million (12.8%) are enrolled in the hottest 20% of schools. In contrast, of the Black and Hispanic students, 0.3 million (11.0%) and 0.7 million (14.4%), respectively, are enrolled in the coolest 20% of schools, while 0.7 million (29.0%) and 1.2 million (25.3%), respectively, are enrolled in the hottest 20% of schools. Thus, Black and Hispanic students are overrepresented in hotter schools and underrepresented in cooler schools, making up a combined 58.7% of students in the hottest 20% of schools (compared to White students who make up 30.1% of students) and only a combined 30.0% of students in the coolest 20% of schools (compared to White students who make up 60.0% of the students enrolled in those schools). These same patterns are found in middle and high schools.

Sensitivity analyses show consistent results, irrespective of how local region, neighborhood socioeconomic status, or home neighborhood are defined. Findings are likewise consistent when modeled with census and temperature data from 2016. Between the earlier and later time periods, the sizes of the effects have trended larger. Full results for all sensitivity analyses are presented in the supplementary materials.

Climate region-specific sensitivity analyses resulted in no contradictory results, although strength and significance of associations varied for all race/ethnicity groups by region. In Table 3, we present model results only for elementary schools and only for the school and neighborhood composition parameters for each of the ten regions. For the Black models, four regions—all in the Western US—failed to achieve statistical significance for either shown parameter; none of these four regions had greater than 7% Black students in their elementary school population. In the remaining five regions, both school and neighborhood demographic coefficients were positive and significant. In the South region, an additional 10% student share of Black students beyond the neighborhood share was associated with an expected 0.25 °C higher temperature while in the Upper Midwest region the same increase was associated with an expected 0.46 °C higher temperature. For both Hispanic and White students, all regions showed significant and consistent model results. The greatest number of Hispanic students are found in the West region, where a 10% increase in the Hispanic student share beyond the neighborhood share is associated with an 0.17 °C increase in temperature, while in the Ohio Valley region, home to the greatest number of White students, a 10% increase in the White student share beyond the neighborhood share is associated with a 0.44 °C decrease in temperature (please see supplementary materials for additional parameters and school levels).

**Table 3:** Regional OLS results, student/population race/ethnicity differences in September afternoon school LST for elementary schools

Climate region	Model parameter	Black	Hispanic	White
West				
	Student count (%)	130,229 (5.4%)	1,375,938 (56.9%)	1,446,611 (20.4%)
	School demographic residual	− 0.16 (− 0.33, 0.00)	**0.17 (0.11, 0.23)**	− **0.28 (**− **0.35,** − **0.20)**
	Catchment demographics	**0.15 (0.04, 0.27)**	**0.31 (0.28, 0.35)**	− **0.41 (**− **0.45,** − **0.37)**
Northwest				
	Student count	27,132 (3.8%)	179,211 (24.6%)	394,137 (54.7%)
	School demographic residual	0.22 (− 0.02, 045)	**0.37 (0.27, 0.47)**	− **0.34 (**− **0.43,** − **0.25)**
	Catchment demographics	**1.04 (0.79, 1.29)**	**0.36 (0.26, 0.45)**	− **0.40 (**− **0.47,** − **0.33)**
Southwest				
	Student count	40,902 (4.1%)	389,627 (39.4%)	464,358 (46.9%)
	School demographic residual	0.09 (− 0.11, 0.30)	**0.09 (0.03, 0.16)**	− **0.16 (-0.22,** − **0.09)**
	Catchment demographics	**0.56 (0.36, 0.76)**	**0.25 (0.20, 0.30)**	− **0.36 (-0.40,** − **0.31)**
Northern Rockies and Plains				
	Student count	14,616 (6.9%)	35,962 (17.0%)	138,039 (65.3%)
	School demographic residual	**0.28 (0.08, 0.49)**	**0.33 (0.22, 0.43)**	− **0.34 (**− **0.41,** − **0.26)**
	Catchment demographics	− 0.08 (− 0.23, 0.08)	**0.18 (0.08, 0.28)**	− **0.15 (**− **0.23,** − **0.07)**
South				
	Student count	429,186 (15.6%)	1,104,895 (40.2%)	949,406 (34.5%)
	School demographic residual	**0.25 (0.20, 0.31)**	**0.20 (0.16, 0.25)**	− **0.31 (**− **0.34,** − **0.27)**
	Catchment demographics	**0.19 (0.16, 0.23)**	**0.26 (0.23, 0.29)**	− **0.37 (**− **0.39,** − **0.34)**
Upper Midwest				
	Student count	93,045 (8.6%)	109,325 (10.1%)	755,101 (69.9%)
	School demographic residual	**0.46 (0.38, 0.55)**	**0.35 (0.26, 0.44)**	− **0.38 (**− **0.43,** − **0.33)**
	Catchment demographics	**0.54 (0.47, 0.62)**	**0.48 (0.38, 0.59)**	− **0.37 (**− **0.42,** − **0.33)**
Ohio Valley				
	Student count	382,198 (14.1%)	406,754 (15.0%)	1,660,365 (61.4%)
	School demographic residual	**0.37 (0.32, 0.41)**	**0.25 (0.20, 0.31)**	− **0.44 (**− **0.47,** − **0.41)**
	Catchment demographics	**0.23 (0.20, 0.26)**	**0.31 (0.27, 0.34)**	− **0.40 (**− **0.43,** − **0.38)**
Southeast				
	Student count	823,651 (26.8%)	705,568 (22.9%)	1,247,625 (40.5%)
	School demographic residual	**0.26 (0.22, 0.30)**	**0.05 (0.00, 0.10)**	− **0.30 (**− **0.34,** − **0.27)**
	Catchment demographics	**0.21 (0.18, 0.23)**	**0.19 (0.15, 0.23)**	− **0.34 (**− **0.37,** − **0.32)**
Northeast				
	Student count	366,889 (13.9%)	526,992 (20.0%)	1,446,611 (54.8%)
	School demographic residual	**0.31 (0.24, 0.38)**	**0.18 (0.12, 0.23)**	− **0.42 (**− **0.46,** − **0.38)**
	Catchment demographics	**0.34 (0.31, 0.38)**	**0.50 (0.45, 0.54)**	− **0.56 (**− **0.59,** − **0.54)**

Student counts are the number and the percent of all regional students in parentheses. Parameter coefficients are shown with confidence intervals in parentheses. Significant associations are shown in bold. Models include neighborhood poverty and MSA-level fixed effects; region and race/ethnicity models run independently

## Discussion

This study revealed important disparities in children’s heat exposure at school, with schools located in neighborhoods with greater concentrations of Black and Hispanic residents and higher poverty having warmer temperatures, and schools with higher shares of Black and Hispanic students even worse off. This is in line with previous research showing disparities in heat based on both neighborhood demographics and socioeconomic status, but we present new evidence of the additional burden faced by students in schools with larger Black or Hispanic student populations relative to their surrounding neighborhood. For example, while the areas around elementary schools show a 0.25 °C and 0.29 °C increase in September afternoon LST for every 10% increase in Black and Hispanic catchment area residents, respectively, they show an additional 0.29 °C increase in September afternoon LST for Black student populations 10% in excess of the local catchment area and 0.20 °C for Hispanic students. In addition, yet another 0.55 °C and 0.57 °C, respectively, increase is expected with a 1% increase in the local poverty rate. In contrast, yet in line with previous research, schools are 0.42 °C cooler with 10% more White residents in the catchment, and an additional 0.35 °C cooler for every 10% more White students than the catchment. Poverty remains a predictor in these models, although to a lesser extent.

Greater proportions of Black and Hispanic students attend the warmest schools than White students. This is partly due to national-level geographic sorting, with Hispanic populations centered in the southwestern US and Black population centered in the southeastern US. It is also partly due to the greater likelihood that Black and Hispanic families will live in neighborhoods with higher poverty rates, which is independently associated with higher school temperature [32]. This results in a significant burden on Black and Hispanic students. As discussed above, a 1.5 °C increase in average temperature was associated by Roach and Whitney with a 10.5% reduction in test scores; our findings show that such a temperature increase is expected to be exceeded between a school with 10% Black students in a 10% Black neighborhood and a school with 60% Black students in a 40% Black neighborhood with identical neighborhood poverty [7]. Given that even a small increase in heat exposure can result in measurable decreases in test scores, school-based heat exposure becomes another source of disadvantage for already burdened communities.

On average, schools have more Black and Hispanic students and fewer White students than the surrounding neighborhoods. This could result from either differences in age distribution between populations (e.g., older average White populations) or White children being more likely to attend schools outside of the neighborhood (e.g., magnet schools) or dataset (e.g., private schools); we expect both reasons to be present in the dataset in different places [33]. Candipan [33] examined the gap in demographic composition between neighborhoods and schools in the context of neighborhood change and found that such gaps are most likely to be found in economically ascendant neighborhoods, where White families with resources move into rising neighborhoods but send their children to private schools. In this context, our findings that show both this gap and higher neighborhood poverty to be associated with higher school temperatures may represent two different mechanisms by which these disparities arise. Namely, the well-documented association between disparities in distribution of green space, tree coverage, and impervious surface and income driving higher temperatures in poor neighborhoods on the one hand and disinvestment in local schools by incoming wealthy residents on the other. Both mechanisms should be further investigated.

Regional analysis of these data show consistency in racial disparities even with differences in effect size between regions. Some of this is due to small population size, such as for Black students in the Northern Rockies and Great Plains (e.g., Montana, Nebraska, North Dakota, South Dakota, and Wyoming). Yet the Southwest (e.g., Arizona, Colorado, New Mexico, and Utah) has close to equal representation of White and Hispanic school children, and for both groups, there is no longer a significant association with the school demographic residual by high school. This may have to do with regionally-specific residential segregation patterns or the size of the catchment area for large secondary schools. Despite such differences, the general consistency in these findings across regions and school levels, given vastly different racial and ethnic composition and climate conditions across the US, is notable and suggests that underlying structural drivers are consistent throughout the country.

Heat in the schoolyard is a relevant exposure for school-aged youth. Physical activity is good for kids, from both a health and learning perspective [34, 35]. This, combined with study results showing that children participate in less school-based physical activity in the heat, suggests that understanding disparities in heat exposure is important [2, 3]. While only a portion of each school day will be spent outside in the schoolyard, it is still a necessary environment to traverse, and thus not only before- and after-school play as well as any recess and outdoor physical education, but also the commute to school and outdoor learning opportunities will be impacted by outdoor temperatures, including outcomes such as general discomfort, dehydration, and heat stress and illness. Some schools have functioning indoor air conditioning, although we know of no national-level dataset with that information, and anecdotal evidence suggests that not all schools where it would be beneficial have such resources available [36]. In schools without air conditioning, indoor thermal conditions are likely to mirror outdoor temperatures, thereby impeding learning and health outcomes for students on hot days [18].

Findings of temperature inequity at the school level present an important opportunity for communities. Schools are important social and geographic locations within neighborhoods and represent both opportunities to bring communities together and to provide resources. Schools and their surrounds could be targeted for nature-based heat remediation efforts that would simultaneously address the identified school heat problem and be a showcase for the range of efforts available in other neighborhood locations. Because the US represents such a broad range of climate areas, these solutions will need to be locally tailored. Broadly applicable interventions may involve the planting of local tree species that are appropriate for the climate and can provide shade when mature, or swapping out sod for local wildflower or rain gardens that stabilize near-surface humidity and manage runoff [37]. Engineered shade solutions have also been applied broadly in schoolyards [38]. Using schools as demonstration points may encourage residents to try to implement temperature regulation strategies in their own private or other public spaces. An additional reason why schools are relevant is that funding streams may be available both via government (e.g., safety-based improvements to school playgrounds) and other (e.g., non-profits that fund and build school gardens) mechanisms. Thus, community-led or demonstration projects may have more funding opportunities at schools than in other neighborhood locations.

This study has important strengths. We used a national sample of schools, representing students in each of the 48 contiguous states and Washington, DC. By including MSA-level fixed effects we controlled for broader geographic racial and ethnic patterning in the US, and present findings for school locations relative to local conditions. We also present findings that are robust to alternative spatial units.

Despite strengths, there are some limitations that further research could attempt to address. First, LST is collected only for cloudless pixels. Therefore, in climates with many cloudy September afternoons where the clouds are preferentially located in certain places, if the clouds are associated with lower temperatures, then the observed LST is an overestimation. This could be an issue in places with sudden topographic shifts (e.g., Salt Lake City) or significant coastlines. In general, we believe that controlling for the local area addressed this issue, but more information would help to improve the data available with which to understand neighborhood level thermal inequities. Second, there may be bias in which schools report to the federally mandated NCES survey programs, and there may be bias associated with districts that choose to be entirely open enrollment schools. However, our sample has good regional coverage mitigating those biases. Third, we do not account for differences in the places where children are likely to spend time and play, such as playing fields and playgrounds, and places where children will pass through, such as parking lots. However, our school temperature measurement was made of the nine 1-km pixels surrounding the school, averaged across all the space, and we suggest that the school itself being surrounded by higher temperatures may be detrimental to students regardless of where the highest temperatures are concentrated. Relatedly, we do not have the capacity to control for schools with or without air conditioning, which will significantly impact the association between indoor and outdoor temperatures [18]. And finally, we do not include rural schools. Schools in rural locations are not impacted by modifiable conditions associated with urban heat islands and thus are not experiencing the same level of heat burden as those in cities. Nonetheless, temperatures throughout the nation are increasing and it is unclear what associations with temperature these schools may yet possess.

## Conclusions

Our findings suggest that programs and policies are needed that privilege schools in neighborhoods with large Black and Hispanic populations, and schools with large Black and Hispanic student populations, in addressing outdoor and indoor heat. This study can provide a baseline against which to compare changes over time. This would include both worsening heat due to climate change as well as the impact of mitigation efforts. Researchers are working to understand contributors to heat stress in school settings, and many schools, districts, and municipalities are turning such research into action both in the classroom and on the playground [39]. Such efforts, targeted towards the schools and students with the greatest need, represent an opportunity to provide all students with the best conditions to succeed and live their healthiest lives.

## Supplementary Information

Below is the link to the electronic supplementary material.

Supplementary file1 (DOCX 131 KB)

## Data Availability

Data is available upon reasonable request to the corresponding author.
