# Supplementary material for: SchoolHEAT: Racial and Ethnic Inequity in School Temperature
Source: J Urban Health. 2024 Sep 24;101(6):1166–77. doi: 10.1007/s11524-024-00919-y (PMC11652446; doi:10.1007/s11524-024-00919-y)
Supplement: Supplementary file 1 — Supplementary file1 (DOCX 131 KB) [file 11524_2024_919_MOESM1_ESM.docx]

| Supplemental Tables:  **S1: OLS Results, student/population race and ethnicity differences in September afternoon school LST for year 2022 weather and demographics, school catchment as the neighborhood, MSA as the region, and poverty as the neighborhood SES measure** | | | |
| --- | --- | --- | --- |
| **Elementary Schools** | | | |
|  | **Black** | **Hispanic** | **White** |
| **School Demographic Residual** | 0.27*** | 0.23*** | -0.37*** |
|  | 0.23, 0.31 | 0.20, 0.26 | -0.39, -0.34 |
| **Catchment Demographics** | 0.22*** | 0.30*** | -0.43*** |
|  | 0.20, 0.24 | 0.28, 0.32 | -0.45, -0.41 |
| **Catchment Poverty** | 0.63*** | 0.64*** | 0.12*** |
|  | 0.58, 0.67 | 0.60, 0.68 | 0.07, 0.16 |
| **Constant** | 27.33*** | 27.24*** | 31.71*** |
|  | 26.18, 28.49 | 26.10, 28.38 | 30.63, 32.80 |
| **Observations** | 16,403 | 16,403 | 16,403 |
| **R2** | 0.92 | 0.92 | 0.93 |
| **Middle Schools** | | | |
|  | **Black** | **Hispanic** | **White** |
| **School Demographic Residual** | 0.27*** | 0.23*** | -0.36*** |
|  | 0.21, 0.32 | 0.19, 0.27 | -0.39, -0.33 |
| **Catchment Demographics** | 0.19*** | 0.28*** | -0.43*** |
|  | 0.16, 0.22 | 0.25, 0.31 | -0.45, -0.41 |
| **Catchment Poverty** | 0.63*** | 0.64*** | 0.09** |
|  | 0.57, 0.69 | 0.59, 0.70 | 0.03, 0.15 |
| **Constant** | 26.92*** | 26.87*** | 31.45*** |
|  | 25.68, 28.15 | 25.65, 28.09 | 30.27, 32.63 |
| **Observations** | 9,252 | 9,252 | 9,252 |
| **R2** | 0.94 | 0.94 | 0.95 |
| **High Schools** | | | |
|  | **Black** | **Hispanic** | **White** |
| **School Demographic Residual** | 0.41*** | 0.21*** | -0.33*** |
|  | 0.31, 0.51 | 0.12, 0.29 | -0.39, -0.27 |
| **Catchment Demographics** | 0.33*** | 0.40*** | -0.50*** |
|  | 0.27, 0.40 | 0.34, 0.47 | -0.55, -0.46 |
| **Catchment Poverty** | 0.61*** | 0.71*** | 0.09 |
|  | 0.49, 0.74 | 0.59, 0.82 | -0.03, 0.22 |
| **Constant** | 26.79*** | 26.61*** | 31.89*** |
|  | 25.40, 28.18 | 25.23, 28.00 | 30.49, 33.29 |
| **Observations** | 3,584 | 3,584 | 3,584 |
| **R2** | 0.93 | 0.93 | 0.94 |
|  |  |  |  |
| **S2: OLS Results, student/population race and ethnicity differences in September afternoon school LST for year 2022 weather and demographics, the Voronoi neighborhood as the neighborhood, MSA as the region, and poverty as the neighborhood SES measure** | | | |
| **Elementary Schools** | | | |
|  | **Black** | **Hispanic** | **White** |
| **School Demographic Residual** | 0.29*** | 0.24*** | -0.36*** |
|  | 0.25, 0.32 | 0.21, 0.27 | -0.38, -0.34 |
| **Voronoi Neighborhood Demographics** | 0.18*** | 0.27*** | -0.40*** |
|  | 0.16, 0.20 | 0.25, 0.29 | -0.42, -0.39 |
| **Voronoi Neighborhood Poverty** | 0.62*** | 0.60*** | 0.15*** |
|  | 0.58, 0.66 | 0.56, 0.63 | 0.11, 0.19 |
| **Constant** | 27.37*** | 27.34*** | 31.63*** |
|  | 26.23, 28.52 | 26.21, 28.48 | 30.54, 32.71 |
| **Observations** | 16,403 | 16,403 | 16,403 |
| **R2** | 0.92 | 0.92 | 0.93 |
| **Middle Schools** | | | |
|  | **Black** | **Hispanic** | **White** |
| **School Demographic Residual** | 0.27*** | 0.25*** | -0.36*** |
|  | 0.22, 0.32 | 0.22, 0.29 | -0.39, -0.33 |
| **Voronoi Neighborhood Demographics** | 0.16*** | 0.25*** | -0.41*** |
|  | 0.13, 0.19 | 0.22, 0.28 | -0.43, -0.39 |
| **Voronoi Neighborhood Poverty** | 0.60*** | 0.59*** | 0.13*** |
|  | 0.54, 0.63 | 0.54, 0.63 | 0.08, 0.19 |
| **Constant** | 26.88*** | 26.90*** | 31.29*** |
|  | 25.65, 28.11 | 25.69, 28.12 | 30.11, 32.47 |
| **Observations** | 9,252 | 9,252 | 9,252 |
| **R2** | 0.94 | 0.94 | 0.94 |
| **High Schools** | | | |
|  | **Black** | **Hispanic** | **White** |
| **School Demographic Residual** | 0.38*** | 0.28*** | -0.35*** |
|  | 0.30, 0.46 | 0.21, 0.36 | -0.40, -0.31 |
| **Voronoi Neighborhood Demographics** | 0.29*** | 0.36*** | -0.48*** |
|  | 0.23, 0.36 | 0.29, 0.42 | -0.53, -0.43 |
| **Voronoi Neighborhood Poverty** | 0.57*** | 0.62*** | 0.10 |
|  | 0.46, 0.67 | 0.52, 0.71 | -0.01, 0.22 |
| **Constant** | 26.82*** | 26.69*** | 31.82*** |
|  | 25.43, 28.21 | 25.31, 28.08 | 30.41, 33.23 |
| **Observations** | 3,584 | 3,584 | 3,584 |
| **R2** | 0.92 | 0.92 | 0.93 |

| **S3: OLS Results, student/population race and ethnicity differences in September afternoon school LST for year 2022 weather and demographics, school catchment as the neighborhood, state as the region, and poverty as the neighborhood SES measure** | | | |
| --- | --- | --- | --- |
| **Elementary Schools** | | | |
|  | **Black** | **Hispanic** | **White** |
| **School Demographic Residuals** | 0.31*** | 0.28*** | -0.35*** |
|  | 0.26, 0.36 | 0.24, 0.32 | -0.38, -0.32 |
| **Catchment Demographics** | 0.31*** | 0.51*** | -0.55*** |
|  | 0.28, 0.33 | 0.48, 0.53 | -0.57, -0.53 |
| **Catchment Poverty** | 0.57*** | 0.48*** | -0.01 |
|  | 0.52, 0.63 | 0.44, 0.53 | -0.07, 0.04 |
| **Constant** | 29.46*** | 30.60*** | 34.95*** |
|  | 29.06, 29.87 | 30.21, 30.99 | 34.55, 35.35 |
| **Observations** | 16,403 | 16,403 | 16,403 |
| **R2** | 0.84 | 0.85 | 0.87 |
| **Middle Schools** | | | |
|  | **Black** | **Hispanic** | **White** |
| **School Demographic Residuals** | 0.37*** | 0.33*** | -0.36*** |
|  | 0.29, 0.44 | 0.27, 0.38 | -0.40, -0.32 |
| **Catchment Demographics** | 0.36*** | 0.52*** | -0.59*** |
|  | 0.32, 0.39 | 0.49, 0.55 | -0.62, -0.57 |
| **Catchment Poverty** | 0.55*** | 0.53*** | -0.08* |
|  | 0.48, 0.63 | 0.46, 0.60 | -0.15, -00.00 |
| **Constant** | 29.14*** | 30.07*** | 35.26*** |
|  | 28.64, 29.65 | 29.58, 30.55 | 34.74, 35.78 |
| **Observations** | 9,252 | 9,252 | 9,252 |
| **R2** | 0.86 | 0.87 | 0.88 |
| **High Schools** | | | |
|  | **Black** | **Hispanic** | **White** |
| **School Demographic Residuals** | 0.45*** | 0.36*** | -0.30*** |
|  | 0.32, 0.58 | 0.25, 0.47 | -0.38, -0.22 |
| **Catchment Demographics** | 0.47*** | 0.68*** | -0.61*** |
|  | 0.39, 0.55 | 0.62, 0.74 | -0.66, -0.56 |
| **Catchment Poverty** | 0.38*** | 0.35*** | -0.17* |
|  | 0.24, 0.52 | 0.22, 0.48 | -0.31, -0.03 |
| **Constant** | 29.26*** | 30.22*** | 35.58*** |
|  | 28.59, 29.94 | 29.58, 30.87 | 34.81, 36.35 |
| **Observations** | 3,584 | 3,584 | 3,584 |
| **R2** | 0.83 | 0.84 | 0.85 |
|  |  |  |  |
|  |  |  |  |

| **S4: OLS Results, student/population race and ethnicity differences in September afternoon school LST for year 2022 weather and demographics, the Voronoi neighborhood as the neighborhood, state as the region, and poverty as the neighborhood SES measure** | | | |
| --- | --- | --- | --- |
| **Elementary Schools** | | | |
|  | **Black** | **Hispanic** | **White** |
| **School Demographic Residuals** | 0.34*** | 0.32*** | -0.38*** |
|  | 0.29, 0.38 | 0.28, 0.35 | -0.41, -0.36 |
| **Voronoi Neighborhood Demographics** | 0.27*** | 0.50*** | -0.38*** |
|  | 0.24, 0.29 | 0.47, 0.52 | -0.41, -0.36 |
| **Voronoi Neighborhood Poverty** | 0.57*** | 0.44*** | -0.01 |
|  | 0.52, 0.61 | 0.40, 0.48 | -0.05, 0.04 |
| **Constant** | 29.40*** | 30.63*** | 35.00*** |
|  | 29.00, 29.80 | 30.25, 31.02 | 34.59, 35.41 |
| **Observations** | 16,403 | 16,403 | 16,403 |
| **R2** | 0.84 | 0.85 | 0.87 |
| **Middle Schools** | | | |
|  | **Black** | **Hispanic** | **White** |
| **School Demographic Residuals** | 0.38*** | 0.38*** | -0.41*** |
|  | 0.32, 0.45 | 0.33, 0.43 | -0.45, -0.38 |
| **Voronoi Neighborhood Demographics** | 0.32*** | 0.50*** | -0.41*** |
|  | 0.29, 0.36 | 0.47, 0.53 | -0.45, -0.38 |
| **Voronoi Neighborhood Poverty** | 0.51*** | 0.45*** | -0.08* |
|  | 0.45, 0.58 | 0.39, 0.52 | -0.15, -0.01 |
| **Constant** | 29.14*** | 30.19*** | 35.35*** |
|  | 28.63, 29.64 | 29.70, 30.68 | 34.82, 35.88 |
| **Observations** | 9,252 | 9,252 | 9,252 |
| **R2** | 0.86 | 0.87 | 0.88 |
| **High Schools** | | | |
|  | **Black** | **Hispanic** | **White** |
| **School Demographic Residuals** | 0.41*** | 0.45*** | -0.34*** |
|  | 0.31, 0.51 | 0.36, 0.54 | -0.40, -0.28 |
| **Voronoi Neighborhood Demographics** | 0.41*** | 0.65*** | -0.34*** |
|  | 0.34, 0.49 | 0.58, 0.71 | -0.40, -0.28 |
| **Voronoi Neighborhood Poverty** | 0.40*** | 0.34*** | -0.12 |
|  | 0.28, 0.53 | 0.22, 0.45 | -0.24, 0.01 |
| **Constant** | 29.24*** | 30.32*** | 35.56*** |
|  | 28.58, 29.91 | 29.68, 30.97 | 34.77, 36.34 |
| **Observations** | 3,584 | 3,584 | 3,584 |
| **R2** | 0.83 | 0.84 | 0.85 |
|  |  |  |  |
|  |  |  |  |

| **S5: OLS Results, student/population race and ethnicity differences in September afternoon school LST for year 2022 weather and demographics, school catchment as the neighborhood, the 5-mile buffer as the region, and poverty as the neighborhood SES measure** | | | |
| --- | --- | --- | --- |
| **Elementary Schools** | | | |
|  | **Black** | **Hispanic** | **White** |
| **School Demographic Residuals** | 0.04*** | 0.09*** | -0.09*** |
|  | 0.02, 0.06 | 0.07, 0.10 | -0.11, -0.08 |
| **Catchment Demographics** | -0.01* | 0.05*** | -0.03*** |
|  | -0.02, -0.00 | 0.04, 0.06 | -0.04, -0.03 |
| **Catchment Poverty** | 0.30*** | 0.27*** | 0.24*** |
|  | 0.28, 0.32 | 0.25, 0.29 | 0.22, 0.26 |
| **5-mile Average LST** | 0.96*** | 0.95*** | 0.96*** |
|  | 0.96, 0.97 | 0.95, 0.95 | 0.95, 0.96 |
| **Constant** | 1.45*** | 1.77*** | 1.99*** |
|  | 1.37, 1.53 | 1.68, 1.86 | 1.86, 2.13 |
| **Observations** | 16,403 | 16,403 | 16,403 |
| **R2** | 0.98 | 0.98 | 0.98 |
| **Middle Schools** | | | |
|  | **Black** | **Hispanic** | **White** |
| **School Demographic Residuals** | 0.04** | 0.09*** | -0.10*** |
|  | 0.01, 0.07 | 0.07, 0.11 | -0.11, -0.08 |
| **Catchment Demographics** | -0.01 | 0.06*** | -0.04*** |
|  | -0.02, 0.01 | 0.05, 0.07 | -0.05, -0.03 |
| **Catchment Poverty** | 0.26*** | 0.24*** | 0.20*** |
|  | 0.23, 0.29 | 0.21, 0.26 | 0.17, 0.23 |
| **5-mile Average LST** | 0.97*** | 0.96*** | 0.96*** |
|  | 0.97, 0.97 | 0.95, 0.96 | 0.95, 0.96 |
| **Constant** | 1.28*** | 1.63*** | 1.97*** |
|  | 1.19, 1.38 | 1.52, 1.75 | 1.81, 2.14 |
| **Observations** | 9,252 | 9,252 | 9,252 |
| **R2** | 0.98 | 0.98 | 0.98 |
| **High Schools** | | | |
|  | **Black** | **Hispanic** | **White** |
| **School Demographic Residuals** | 0.06** | 0.10*** | -0.09*** |
|  | 0.02, 0.11 | 0.05, 0.14 | -0.12, -0.06 |
| **Catchment Demographics** | 0.02 | 0.05*** | -0.05*** |
|  | -0.00, 0.05 | 0.02, 0.07 | -0.06, -0.03 |
| **Catchment Poverty** | 0.30*** | 0.31** | 0.25** |
|  | 0.25, 0.35 | 0.26, 0.36 | 0.20, 0.30 |
| **5-mile Average LST** | 0.96*** | 0.95*** | 0.95*** |
|  | 0.95, 0.96 | 0.94, 0.95 | 0.94, 0.95 |
| **Constant** | 1.57*** | 1.81*** | 2.30*** |
|  | 1.41, 1.72 | 1.63, 1.99 | 1.99, 2.61 |
| **Observations** | 3,584 | 3,584 | 3,584 |
| **R2** | 0.98 | 0.98 | 0.98 |
|  |  |  |  |
|  |  |  |  |

| **S6: OLS Results, student/population race and ethnicity differences in September afternoon school LST for year 2022 weather and demographics, school catchment as the neighborhood, the 10-mile buffer as the region, and poverty as the neighborhood SES measure** | | | |
| --- | --- | --- | --- |
| **Elementary Schools** | | | |
|  | **Black** | **Hispanic** | **White** |
| **School Demographic Residuals** | 0.13*** | 0.17*** | -0.21*** |
|  | 0.10, 0.16 | 0.14, 0.19 | -0.22, -0.19 |
| **Catchment Demographics** | 0.04*** | 0.11*** | -0.12*** |
|  | 0.03, 0.05 | 0.10, 0.12 | -0.13, -0.11 |
| **Catchment Poverty** | 0.46*** | 0.47*** | 0.34*** |
|  | 0.43, 0.49 | 0.44, 0.49 | 0.32, 0.37 |
| **10-mile Average LST** | 0.95*** | 0.92*** | 0.92*** |
|  | 0.95, 0.95 | 0.92, 0.93 | 0.92, 0.92 |
| **Constant** | 2.01*** | 2.70*** | 3.87*** |
|  | 1.91, 2.12 | 2.58, 2.83 | 3.69, 4.04 |
| **Observations** | 16,403 | 16,403 | 16,403 |
| **R2** | 0.95 | 0.95 | 0.96 |
| **Middle Schools** | | | |
|  | **Black** | **Hispanic** | **White** |
| **School Demographic Residuals** | 0.12*** | 0.19*** | -0.21*** |
|  | 0.08, 0.16 | 0.16, 0.22 | -0.23, -0.18 |
| **Catchment Demographics** | 0.06*** | 0.13*** | -0.14*** |
|  | 0.04, 0.07 | 0.11, 0.14 | -0.15, -0.13 |
| **Catchment Poverty** | 0.42*** | 0.44*** | 0.28*** |
|  | 0.38, 0.46 | 0.40, 0.47 | 0.24, 0.31 |
| **10-mile Average LST** | 0.96*** | 0.93*** | 0.93*** |
|  | 0.96, 0.96 | 0.92, 0.93 | 0.92, 0.93 |
| **Constant** | 1.69*** | 2.48*** | 3.92*** |
|  | 1.56, 1.83 | 2.33, 2.64 | 3.69, 4.15 |
| **Observations** | 9,252 | 9,252 | 9,252 |
| **R2** | 0.96 | 0.96 | 0.96 |
| **High Schools** | | | |
|  | **Black** | **Hispanic** | **White** |
| **School Demographic Residuals** | 0.15*** | 0.18*** | -0.18*** |
|  | 0.08, 0.22 | 0.13, 0.24 | -0.23, -0.14 |
| **Catchment Demographics** | 0.08*** | 0.12*** | -0.14*** |
|  | 0.05, 0.12 | 0.09, 0.15 | -0.16, -0.12 |
| **Catchment Poverty** | 0.47*** | 0.52*** | 0.35*** |
|  | 0.40, 0.54 | 0.45, 0.58 | 0.28, 0.42 |
| **10-mile Average LST** | 0.94*** | 0.92*** | 0.91*** |
|  | 0.94, 0.95 | 0.91, 0.93 | 0.90, 0.92 |
| **Constant** | 1.95*** | 2.54*** | 4.09*** |
|  | 1.74, 2.17 | 2.29, 2.79 | 3.68, 4.50 |
| **Observations** | 3,584 | 3,584 | 3,584 |
| **R2** | 0.95 | 0.95 | 0.95 |
|  |  |  |  |
|  |  |  |  |

| **S7: OLS Results, student/population race and ethnicity differences in September afternoon school LST for year 2022 weather and demographics, school catchment as the neighborhood, the 25-mile buffer as the region, and poverty as the neighborhood SES measure** | | | |
| --- | --- | --- | --- |
| **Elementary Schools** | | | |
|  | **Black** | **Hispanic** | **White** |
| **School Demographic Residuals** | 0.23*** | 0.24*** | -0.31*** |
|  | 0.19, 0.26 | 0.21, 0.27 | -0.33, -0.29 |
| **Catchment Demographics** | 0.19*** | 0.21*** | -0.28*** |
|  | 0.17, 0.20 | 0.19, 0.22 | -0.29, -0.27 |
| **Catchment Poverty** | 0.52*** | 0.61*** | 0.31*** |
|  | 0.48, 0.55 | 0.58, 0.64 | 0.28, 0.34 |
| **25-mile Average LST** | 0.94*** | 0.88*** | 0.87*** |
|  | 0.94, 0.94 | 0.88, 0.89 | 0.87, 0.88 |
| **Constant** | 2.76*** | 4.13*** | 6.96*** |
|  | 2.63, 2.90 | 3.97, 4.28 | 6.75, 7.17 |
| **Observations** | 16,403 | 16,403 | 16,403 |
| **R2** | 0.92 | 0.92 | 0.93 |
| **Middle Schools** | | | |
|  | **Black** | **Hispanic** | **White** |
| **School Demographic Residuals** | 0.22*** | 0.31*** | -0.33*** |
|  | 0.17, 0.27 | 0.27, 0.35 | -0.36, -0.30 |
| **Catchment Demographics** | 0.23*** | 0.26*** | -0.33*** |
|  | 0.21, 0.25 | 0.24, 0.28 | -0.34, -0.31 |
| **Catchment Poverty** | 0.47*** | 0.60*** | 0.21*** |
|  | 0.42, 0.52 | 0.55, 0.64 | 0.16, 0.26 |
| **25-mile Average LST** | 0.95*** | 0.89*** | 0.88*** |
|  | 0.95, 0.96 | 0.88, 0.89 | 0.87, 0.88 |
| **Constant** | 2.28*** | 3.88*** | 7.29*** |
|  | 2.11, 2.46 | 3.68, 4.08 | 7.02, 7.56 |
| **Observations** | 9,252 | 9,252 | 9,252 |
| **R2** | 0.93 | 0.93 | 0.94 |
| **High Schools** | | | |
|  | **Black** | **Hispanic** | **White** |
| **School Demographic Residuals** | 0.29*** | 0.28*** | -0.29*** |
|  | 0.21, 0.38 | 0.20, 0.35 | -0.34, -0.24 |
| **Catchment Demographics** | 0.24*** | 0.27*** | -0.32*** |
|  | 0.20, 0.29 | 0.23, 0.31 | -0.35, -0.29 |
| **Catchment Poverty** | 0.53*** | 0.67*** | 0.30*** |
|  | 0.44, 0.62 | 0.59, 0.75 | 0.21, 0.38 |
| **25-mile Average LST** | 0.93*** | 0.88*** | 0.87*** |
|  | 0.92, 0.94 | 0.87, 0.89 | 0.86, 0.88 |
| **Constant** | 2.44*** | 3.67*** | 7.17*** |
|  | 2.17, 2.71 | 3.36, 3.98 | 6.68, 7.65 |
| **Observations** | 3,584 | 3,584 | 3,584 |
| **R2** | 0.92 | 0.92 | 0.93 |
|  |  |  |  |
|  |  |  |  |

| **S8: OLS Results, student/population race and ethnicity differences in September afternoon school LST for year 2022 weather and demographics, the Voronoi neighborhood as the neighborhood, the 5-mile buffer as the region, and poverty as the neighborhood SES measure** | | | |
| --- | --- | --- | --- |
| **Elementary Schools** | | | |
|  | **Black** | **Hispanic** | **White** |
| **School Demographic Residuals** | 0.04*** | 0.08*** | -0.08*** |
|  | 0.03, 0.06 | 0.07, 0.10 | -0.09, -0.07 |
| **Voronoi Neighborhood Demographics** | -0.02*** | 0.04*** | -0.02*** |
|  | -0.03, -0.01 | 0.03, 0.05 | -0.03, -0.01 |
| **Voronoi Neighborhood Poverty** | 0.29*** | 0.26*** | 0.25*** |
|  | 0.27, 0.31 | 0.25, 0.28 | 0.23, 0.27 |
| **5-mile Average LST** | 0.96*** | 0.95*** | 0.95*** |
|  | 0.96, 0.96 | 0.95, 0.95 | 0.95, 0.96 |
| **Constant** | 1.51*** | 1.79*** | 1.92*** |
|  | 1.44, 1.59 | 1.70, 1.88 | 1.78, 2.05 |
| **Observations** | 16,403 | 16,403 | 16,403 |
| **R2** | 0.98 | 0.98 | 0.98 |
| **Middle Schools** | | | |
|  | **Black** | **Hispanic** | **White** |
| **School Demographic Residuals** | 0.04*** | 0.09*** | -0.08*** |
|  | 0.02, 0.06 | 0.07, 0.11 | -0.10, -0.07 |
| **Voronoi Neighborhood Demographics** | -0.01* | 0.05*** | -0.03*** |
|  | -0.03, -00.00 | 0.04, 0.06 | -0.04, -0.02 |
| **Voronoi Neighborhood Poverty** | 0.26*** | 0.23*** | 0.21*** |
|  | 0.24, 0.28 | 0.21, 0.26 | 0.19, 0.24 |
| **5-mile Average LST** | 0.97*** | 0.95*** | 0.96*** |
|  | 0.96, 0.97 | 0.95, 0.96 | 0.95, 0.96 |
| **Constant** | 1.33*** | 1.65*** | 1.88*** |
|  | 1.24, 1.43 | 1.54, 1.76 | 1.71, 2.05 |
| **Observations** | 9,252 | 9,252 | 9,252 |
| **R2** | 0.98 | 0.98 | 0.98 |
| **High Schools** | | | |
|  | **Black** | **Hispanic** | **White** |
| **School Demographic Residuals** | 0.05** | 0.08*** | -0.09*** |
|  | 0.02, 0.09 | 0.05, 0.12 | -0.11, -0.06 |
| **Voronoi Neighborhood Demographics** | 0.01 | 0.04** | -0.03*** |
|  | -0.01, 0.03 | 0.01, 0.06 | -0.05, -0.02 |
| **Voronoi Neighborhood Poverty** | 0.30*** | 0.30*** | 0.27*** |
|  | 0.25, 0.34 | 0.26, 0.34 | 0.23, 0.32 |
| **5-mile Average LST** | 0.95*** | 0.94*** | 0.94*** |
|  | 0.95, 0.96 | 0.94, 0.95 | 0.94, 0.95 |
| **Constant** | 1.59*** | 1.82*** | 2.16*** |
|  | 1.44, 1.74 | 1.63, 2.00 | 1.85, 2.47 |
| **Observations** | 3,584 | 3,584 | 3,584 |
| **R2** | 0.98 | 0.98 | 0.98 |
|  |  |  |  |
|  |  |  |  |

| **S9: OLS Results, student/population race and ethnicity differences in September afternoon school LST for year 2022 weather and demographics, the Voronoi neighborhood as the neighborhood, the 10-mile buffer as the region, and poverty as the neighborhood SES measure** | | | |
| --- | --- | --- | --- |
| **Elementary Schools** | | | |
|  | **Black** | **Hispanic** | **White** |
| **School Demographic Residuals** | 0.13*** | 0.16*** | -0.19*** |
|  | 0.10, 0.15 | 0.14, 0.18 | -0.20, -0.17 |
| **Voronoi Neighborhood Demographics** | 0.02*** | 0.09*** | -0.10*** |
|  | 0.01, 0.04 | 0.08, 0.10 | -0.11, -0.09 |
| **Voronoi Neighborhood Poverty** | 0.46*** | 0.45*** | 0.37*** |
|  | 0.44, 0.49 | 0.43, 0.48 | 0.34, 0.39 |
| **10-mile Average LST** | 0.95*** | 0.92*** | 0.92*** |
|  | 0.94, 0.95 | 0.92, 0.93 | 0.92, 0.92 |
| **Constant** | 2.11*** | 2.72*** | 3.73*** |
|  | 2.00, 2.21 | 2.60, 2.84 | 3.55, 3.91 |
| **Observations** | 16,403 | 16,403 | 16,403 |
| **R2** | 0.95 | 0.95 | 0.96 |
| **Middle Schools** | | | |
|  | **Black** | **Hispanic** | **White** |
| **School Demographic Residuals** | 0.11*** | 0.18*** | -0.18*** |
|  | 0.08, 0.14 | 0.15, 0.21 | -0.20, -0.16 |
| **Voronoi Neighborhood Demographics** | 0.04*** | 0.11*** | -0.12*** |
|  | 0.02, 0.05 | 0.09, 0.12 | -0.13, -0.11 |
| **Voronoi Neighborhood Poverty** | 0.43*** | 0.43*** | 0.31*** |
|  | 0.40, 0.47 | 0.40, 0.46 | 0.28, 0.35 |
| **10-mile Average LST** | 0.96*** | 0.93*** | 0.92*** |
|  | 0.95, 0.96 | 0.92, 0.93 | 0.92, 0.93 |
| **Constant** | 1.77*** | 2.50*** | 3.75*** |
|  | 1.64, 1.90 | 2.35, 2.65 | 3.52, 3.98 |
| **Observations** | 9,252 | 9,252 | 9,252 |
| **R2** | 0.96 | 0.96 | 0.96 |
| **High Schools** | | | |
|  | **Black** | **Hispanic** | **White** |
| **School Demographic Residuals** | 0.14*** | 0.18*** | -0.19*** |
|  | 0.09, 0.20 | 0.14, 0.23 | -0.22, -0.15 |
| **Voronoi Neighborhood Demographics** | 0.06*** | 0.10*** | -0.12*** |
|  | 0.03, 0.10 | 0.07, 0.13 | -0.14, -0.10 |
| **Voronoi Neighborhood Poverty** | 0.44*** | 0.48*** | 0.36*** |
|  | 0.38, 0.50 | 0.42, 0.53 | 0.30, 0.42 |
| **10-mile Average LST** | 0.94*** | 0.92*** | 0.91*** |
|  | 0.93, 0.95 | 0.91, 0.93 | 0.90, 0.92 |
| **Constant** | 2.00*** | 2.57*** | 3.91*** |
|  | 1.79, 2.21 | 2.32, 2.82 | 3.49, 4.32 |
| **Observations** | 3,584 | 3,584 | 3,584 |
| **R2** | 0.95 | 0.95 | 0.96 |
|  |  |  |  |
|  |  |  |  |

| **S10: OLS Results, student/population race and ethnicity differences in September afternoon school LST for year 2022 weather and demographics, the Voronoi neighborhood as the neighborhood, the 25-mile buffer as the region, and poverty as the neighborhood SES measure** | | | |
| --- | --- | --- | --- |
| **Elementary Schools** | | | |
|  | **Black** | **Hispanic** | **White** |
| **School Demographic Residuals** | 0.22*** | 0.23*** | -0.29*** |
|  | 0.19, 0.25 | 0.21, 0.26 | -0.31, -0.27 |
| **Voronoi Neighborhood Demographics** | 0.16*** | 0.19*** | -0.25*** |
|  | 0.14, 0.17 | 0.17, 0.20 | -0.27, -0.24 |
| **Voronoi Neighborhood Poverty** | 0.54*** | 0.59*** | 0.34*** |
|  | 0.51, 0.57 | 0.56, 0.62 | 0.31, 0.37 |
| **25-mile Average LST** | 0.93*** | 0.88*** | 0.87*** |
|  | 0.93, 0.94 | 0.88, 0.89 | 0.87, 0.88 |
| **Constant** | 2.87*** | 4.15*** | 6.84*** |
|  | 2.73, 3.00 | 4.00, 4.31 | 6.63, 7.05 |
| **Observations** | 16,403 | 16,403 | 16,403 |
| **R2** | 0.92 | 0.92 | 0.93 |
| **Middle Schools** | | | |
|  | **Black** | **Hispanic** | **White** |
| **School Demographic Residuals** | 0.21*** | 0.29*** | -0.31*** |
|  | 0.16, 0.25 | 0.26, 0.32 | -0.33, -0.28 |
| **Voronoi Neighborhood Demographics** | 0.20*** | 0.23*** | -0.30*** |
|  | 0.17, 0.22 | 0.21, 0.25 | -0.31, -0.28 |
| **Voronoi Neighborhood Poverty** | 0.52*** | 0.59*** | 0.28*** |
|  | 0.48, 0.56 | 0.55, 0.63 | 0.24, 0.32 |
| **25-mile Average LST** | 0.95*** | 0.89*** | 0.88*** |
|  | 0.94, 0.95 | 0.88, 0.89 | 0.87, 0.88 |
| **Constant** | 2.35*** | 3.89*** | 7.07*** |
|  | 2.18, 2.52 | 3.69, 4.09 | 6.79, 7.34 |
| **Observations** | 9,252 | 9,252 | 9,252 |
| **R2** | 0.93 | 0.93 | 0.94 |
| **High Schools** | | | |
|  | **Black** | **Hispanic** | **White** |
| **School Demographic Residuals** | 0.27*** | 0.29*** | -0.29*** |
|  | 0.20, 0.33 | 0.23, 0.35 | -0.33, -0.25 |
| **Voronoi Neighborhood Demographics** | 0.21*** | 0.23*** | -0.30*** |
|  | 0.16, 0.25 | 0.20, 0.27 | -0.33, -0.27 |
| **Voronoi Neighborhood Poverty** | 0.52*** | 0.62*** | 0.32*** |
|  | 0.45, 0.60 | 0.55, 0.69 | 0.24, 0.40 |
| **25-mile Average LST** | 0.93*** | 0.88*** | 0.87*** |
|  | 0.92, 0.94 | 0.87, 0.89 | 0.86, 0.88 |
| **Constant** | 2.50*** | 3.70*** | 7.00*** |
|  | 2.23, 2.77 | 3.39, 4.02 | 6.51, 7.50 |
| **Observations** | 3,584 | 3,584 | 3,584 |
| **R2** | 0.92 | 0.92 | 0.93 |
|  |  |  |  |
|  |  |  |  |

| **S11: OLS Results, student/population race and ethnicity differences in September afternoon school LST for year 2022 weather and demographics, school catchment as the neighborhood, the MSA as the region, and median household income as the neighborhood SES measure** | | | |
| --- | --- | --- | --- |
| **Elementary Schools** | | | |
|  | **Black** | **Hispanic** | **White** |
| **School Demographic Residuals** | 0.35*** | 0.21*** | -0.38*** |
|  | 0.30, 0.39 | 0.17, 0.24 | -0.40, -0.35 |
| **Catchment Demographics** | 0.27*** | 0.29*** | -0.40*** |
|  | 0.24, 0.29 | 0.27, 0.32 | -0.42, -0.38 |
| **Catchment Median Household Income** | -0.15*** | -0.12*** | -0.04*** |
|  | -0.16, -0.14 | -0.13, -0.11 | -0.06, -0.03 |
| **Constant** | 28.84*** | 28.58*** | 31.79*** |
|  | 27.65, 30.03 | 27.40, 29.76 | 30.68, 32.91 |
| **Observations** | 12,881 | 12,881 | 12,881 |
| **R2** | 0.93 | 0.93 | 0.94 |
| **Middle Schools** | | | |
|  | **Black** | **Hispanic** | **White** |
| **School Demographic Residuals** | 0.41*** | 0.20*** | -0.36*** |
|  | 0.33, 0.48 | 0.15, 0.24 | -0.40, -0.32 |
| **Catchment Demographics** | 0.23*** | 0.26*** | -0.39*** |
|  | 0.19, 0.26 | 0.22, 0.29 | -0.42, -0.37 |
| **Catchment Median Household Income** | -0.15*** | -0.13*** | -0.04*** |
|  | -0.17, -0.13 | -0.14, -0.11 | -0.06, -0.02 |
| **Constant** | 28.25*** | 28.03*** | 31.26*** |
|  | 26.99, 29.52 | 26.76, 29.29 | 30.05, 32.47 |
| **Observations** | 6,755 | 6,755 | 6,755 |
| **R2** | 0.95 | 0.95 | 0.95 |
| **High Schools** | | | |
|  | **Black** | **Hispanic** | **White** |
| **School Demographic Residuals** | 0.64*** | 0.24*** | -0.29*** |
|  | 0.46, 0.81 | 0.13, 0.36 | -0.38, -0.21 |
| **Catchment Demographics** | 0.48*** | 0.35*** | -0.43*** |
|  | 0.37, 0.60 | 0.25, 0.44 | -0.49, -0.37 |
| **Catchment Median Household Income** | -0.05** | -0.05* | -0.01 |
|  | -0.10, -0.01 | -0.09, -0.01 | -0.05, 0.03 |
| **Constant** | 27.39*** | 27.25*** | 31.16*** |
|  | 26.01, 28.78 | 25.85, 28.66 | 29.73, 32.60 |
| **Observations** | 2,412 | 2,412 | 2,412 |
| **R2** | 0.94 | 0.94 | 0.94 |

| **S12: OLS Results, student/population race and ethnicity differences in September afternoon school LST for year 2022 weather and demographics, the Voronoi neighborhood as the neighborhood, the MSA as the region, and median household income as the neighborhood SES measure** | | | |
| --- | --- | --- | --- |
| **Elementary Schools** | | | |
|  | **Black** | **Hispanic** | **White** |
| **School Demographic Residuals** | 0.32*** | 0.20*** | -0.35*** |
|  | 0.28, 0.35 | 0.17, 0.23 | -0.38, -0.33 |
| **Voronoi Neighborhood Demographics** | 0.23*** | 0.28*** | -0.38*** |
|  | 0.21, 0.26 | 0.26, 0.31 | -0.40, -0.37 |
| **Voronoi Median Household Income** | -0.21*** | -0.19*** | -0.09*** |
|  | -0.23, -0.20 | -0.20, -0.17 | -0.10, -0.07 |
| **Constant** | 29.18*** | 28.96*** | 32.07*** |
|  | 28.05, 30.32 | 27.82, 30.09 | 31.01, 33.14 |
| **Observations** | 15,007 | 15,007 | 15,007 |
| **R2** | 0.93 | 0.93 | 0.94 |
| **Middle Schools** | | | |
|  | **Black** | **Hispanic** | **White** |
| **School Demographic Residuals** | 0.29*** | 0.22*** | -0.35*** |
|  | 0.24, 0.34 | 0.18, 0.25 | -0.38, -0.32 |
| **Voronoi Neighborhood Demographics** | 0.20*** | 0.25*** | -0.39*** |
|  | 0.18, 0.23 | 0.22, 0.28 | -0.41, -0.36 |
| **Voronoi Median Household Income** | -0.23*** | -0.20*** | -0.09*** |
|  | -0.25, -0.21 | -0.22, -0.18 | -0.11, -0.06 |
| **Constant** | 28.71*** | 28.53*** | 31.68*** |
|  | 27.50, 29.93 | 27.32, 29.75 | 30.52, 32.83 |
| **Observations** | 8,384 | 8,384 | 8,384 |
| **R2** | 0.94 | 0.94 | 0.95 |
| **High Schools** | | | |
|  | **Black** | **Hispanic** | **White** |
| **School Demographic Residuals** | 0.38*** | 0.25*** | -0.32*** |
|  | 0.29, 0.47 | 0.18, 0.33 | -0.37, -0.27 |
| **Voronoi Neighborhood Demographics** | 0.37*** | 0.35*** | -0.45*** |
|  | 0.30, 0.44 | 0.28, 0.43 | -0.50, -0.40 |
| **Voronoi Median Household Income** | -0.18*** | -0.17*** | -0.07** |
|  | -0.22, -0.14 | -0.21, -0.12 | -0.11, -0.03 |
| **Constant** | 28.46*** | 28.29*** | 32.08*** |
|  | 27.10, 29.82 | 26.91, 29.66 | 30.75, 33.41 |
| **Observations** | 3,193 | 3,193 | 3,193 |
| **R2** | 0.94 | 0.94 | 0.95 |
|  |  |  |  |

| **S13: OLS Results, student/population race and ethnicity differences in September afternoon school LST for year 2022 weather and demographics, school catchment as the neighborhood, the MSA as the region, and median median home value as the neighborhood SES measure** | | | |
| --- | --- | --- | --- |
| **Elementary Schools** | | | |
|  | **Black** | **Hispanic** | **White** |
| **School Demographic Residuals** | 0.40*** | 0.25*** | -0.39*** |
|  | 0.35, 0.45 | 0.21, 0.28 | -0.42, -0.36 |
| **Catchment Demographics** | 0.31*** | 0.33*** | -0.40*** |
|  | 0.28, 0.33 | 0.31, 0.36 | -0.42, -0.39 |
| **Catchment Median Home Value** | -0.03*** | -0.02*** | -0.01*** |
|  | -0.03, -0.03 | -0.02, -0.02 | -0.02, -0.01 |
| **Constant** | 28.21*** | 28.02*** | 31.75*** |
|  | 26.93, 29.50 | 26.74, 29.31 | 30.55, 32.96 |
| **Observations** | 12,769 | 12,769 | 12,769 |
| **R2** | 0.92 | 0.92 | 0.93 |
| **Middle Schools** | | | |
|  | **Black** | **Hispanic** | **White** |
| **School Demographic Residuals** | 0.43*** | 0.23*** | -0.37*** |
|  | 0.35, 0.50 | 0.18, 0.28 | -0.41, -0.33 |
| **Catchment Demographics** | 0.25*** | 0.29*** | -0.40*** |
|  | 0.22, 0.29 | 0.26, 0.32 | -0.42, -0.37 |
| **Catchment Median Home Value** | -0.03*** | -0.02*** | -0.01*** |
|  | -0.03, -0.02 | -0.02, -0.02 | -0.01, -0.01 |
| **Constant** | 27.33*** | 27.19*** | 30.98*** |
|  | 25.93, 28.73 | 25.80, 28.59 | 29.64, 32.32 |
| **Observations** | 6,647 | 6,647 | 6,647 |
| **R2** | 0.94 | 0.94 | 0.95 |
| **High Schools** | | | |
|  | **Black** | **Hispanic** | **White** |
| **School Demographic Residuals** | 0.56*** | 0.26*** | -0.27*** |
|  | 0.36, 0.75 | 0.14, 0.38 | -0.36, -0.19 |
| **Catchment Demographics** | 0.43*** | 0.45*** | -0.44*** |
|  | 0.30, 0.55 | 0.36, 0.54 | -0.50, -0.38 |
| **Catchment Median Home Value** | -0.03*** | -0.02*** | -0.02*** |
|  | -0.03, -0.02 | -0.03, -0.01 | -0.03, -0.01 |
| **Constant** | 27.37*** | 27.17*** | 31.47*** |
|  | 25.97, 28.78 | 25.78, 28.56 | 30.00, 32.95 |
| **Observations** | 2,449 | 2,449 | 2,449 |
| **R2** | 0.93 | 0.93 | 0.93 |
|  |  |  |  |

| **S14: OLS Results, student/population race and ethnicity differences in September afternoon school LST for year 2022 weather and demographics, Voronoi neighborhood as the neighborhood, the MSA as the region, and median median home value as the neighborhood SES measure** | | | |
| --- | --- | --- | --- |
| **Elementary Schools** | | | |
|  | **Black** | **Hispanic** | **White** |
| **School Demographic Residuals** | 0.36*** | 0.22*** | -0.37*** |
|  | 0.32, 0.39 | 0.19, 0.25 | -0.39, -0.35 |
| **Voronoi Neighborhood Demographics** | 0.29*** | 0.33*** | -0.39*** |
|  | 0.26, 0.31 | 0.30, 0.35 | -0.40, -0.37 |
| **Voronoi Neighborhood Median Home Value** | -0.04*** | -0.03*** | -0.02*** |
|  | -0.04, -0.03 | -0.03, -0.03 | -0.02, -0.02 |
| **Constant** | 28.30*** | 28.15*** | 31.80*** |
|  | 27.10, 29.50 | 26.94, 29.35 | 30.68, 32.92 |
| **Observations** | 14,226 | 14,226 | 14,226 |
| **R2** | 0.92 | 0.92 | 0.93 |
| **Middle Schools** | | | |
|  | **Black** | **Hispanic** | **White** |
| **School Demographic Residuals** | 0.35*** | 0.23*** | -0.37*** |
|  | 0.30, 0.40 | 0.19, 0.27 | -0.40, -0.34 |
| **Voronoi Neighborhood Demographics** | 0.25*** | 0.29*** | -0.39*** |
|  | 0.22, 0.28 | 0.26, 0.32 | -0.41, -0.37 |
| **Voronoi Neighborhood Median Home Value** | -0.04*** | -0.03*** | -0.02*** |
|  | -0.04, -0.03 | -0.03, -0.03 | -0.02, -0.01 |
| **Constant** | 27.80*** | 27.70*** | 31.45*** |
|  | 26.59, 29.01 | 26.49, 28.91 | 30.30, 32.59 |
| **Observations** | 7,986 | 7,986 | 7,986 |
| **R2** | 0.94 | 0.94 | 0.95 |
| **High Schools** | | | |
|  | **Black** | **Hispanic** | **White** |
| **School Demographic Residuals** | 0.41*** | 0.25*** | -0.33*** |
|  | 0.32, 0.50 | 0.17, 0.34 | -0.39, -0.28 |
| **Voronoi Neighborhood Demographics** | 0.39*** | 0.40*** | -0.44*** |
|  | 0.32, 0.46 | 0.33, 0.47 | -0.49, -0.40 |
| **Voronoi Neighborhood Median Home Value** | -0.03*** | -0.03*** | -0.02*** |
|  | -0.04, -0.03 | -0.04, -0.02 | -0.03, -0.01 |
| **Constant** | 27.71*** | 27.55*** | 31.83*** |
|  | 26.38, 29.04 | 26.21, 28.89 | 30.50, 33.15 |
| **Observations** | 3,102 | 3,102 | 3,102 |
| **R2** | 0.94 | 0.94 | 0.94 |
|  |  |  |  |

| **S15: OLS Results, student/population race and ethnicity differences in September afternoon school LST for year 2016 weather and demographics, school catchment as the neighborhood, MSA as the region, and poverty as the neighborhood SES measure** | | | |
| --- | --- | --- | --- |
| **Elementary Schools** | | | |
|  | **Black** | **Hispanic** | **White** |
| **School Demographic Residual** | 0.30*** | 0.20*** | -0.35*** |
|  | 0.27, 0.32 | 0.18, 0.22 | -0.36, -0.33 |
| **Catchment Demographics** | 0.21*** | 0.26*** | -0.40*** |
|  | 0.20, 0.22 | 0.24, 0.27 | -0.41, -0.39 |
| **Catchment Poverty** | 0.57*** | 0.56*** | 0.12*** |
|  | 0.54, 0.59 | 0.54, 0.58 | 0.09, 0.15 |
| **Constant** | 27.97*** | 27.95*** | 32.28*** |
|  | 26.81, 29.12 | 26.80, 29.10 | 31.18, 33.37 |
| **Observations** | 36,724 | 36,724 | 36,724 |
| **R2** | 0.9 | 0.9 | 0.91 |
| **Middle Schools** | | | |
|  | **Black** | **Hispanic** | **White** |
| **School Demographic Residual** | 0.30*** | 0.22*** | -0.33*** |
|  | 0.26, 0.33 | 0.19, 0.25 | -0.35, -0.31 |
| **Catchment Demographics** | 0.22*** | 0.27*** | -0.42*** |
|  | 0.20, 0.24 | 0.25, 0.29 | -0.44, -0.41 |
| **Catchment Poverty** | 0.58*** | 0.57*** | 0.10*** |
|  | 0.55, 0.61 | 0.54, 0.60 | 0.07, 0.14 |
| **Constant** | 27.36*** | 27.34*** | 31.87*** |
|  | 26.07, 28.65 | 26.06, 28.63 | 30.63, 33.10 |
| **Observations** | 22,704 | 22,704 | 22,704 |
| **R2** | 0.92 | 0.92 | 0.92 |
| **High Schools** | | | |
|  | **Black** | **Hispanic** | **White** |
| **School Demographic Residual** | 0.42*** | 0.24*** | -0.31*** |
|  | 0.36, 0.47 | 0.18, 0.30 | -0.34, -0.27 |
| **Catchment Demographics** | 0.27*** | 0.33*** | -0.45*** |
|  | 0.24, 0.31 | 0.29, 0.36 | -0.47, -0.42 |
| **Catchment Poverty** | 0.60*** | 0.63*** | 0.13*** |
|  | 0.54, 0.67 | 0.57, 0.68 | 0.06, 0.19 |
| **Constant** | 27.21*** | 27.13*** | 31.84*** |
|  | 25.76, 28.67 | 25.67, 28.59 | 30.42, 33.27 |
| **Observations** | 10,469 | 10,469 | 10,469 |
| **R2** | 0.9 | 0.9 | 0.91 |
|  |  |  |  |
|  |  |  |  |

| **S16: OLS Results, student/population race and ethnicity differences in September afternoon school LST for year 2016 weather and demographics, Voronoi neighborhood as the neighborhood, MSA as the region, and poverty as the neighborhood SES measure** | | | |
| --- | --- | --- | --- |
| **Elementary Schools** | | | |
|  | **Black** | **Hispanic** | **White** |
| **School Demographic Residual** | 0.26*** | 0.18*** | -0.30*** |
|  | 0.24, 0.28 | 0.16, 0.20 | -0.32, -0.29 |
| **Voronoi Neighborhood Demographics** | 0.21*** | 0.27*** | -0.41*** |
|  | 0.20, 0.23 | 0.26, 0.29 | -0.42, -0.40 |
| **Voronoi Neighborhood Poverty** | 0.59*** | 0.57*** | 0.15*** |
|  | 0.56, 0.61 | 0.55, 0.60 | 0.12, 0.17 |
| **Constant** | 27.99*** | 27.97*** | 32.34*** |
|  | 26.84, 29.14 | 26.82, 29.12 | 31.25, 33.43 |
| **Observations** | 36,724 | 36,724 | 36,724 |
| **R2** | 0.90 | 0.90 | 0.91 |
| **Middle Schools** | | | |
|  | **Black** | **Hispanic** | **White** |
| **School Demographic Residual** | 0.28*** | 0.20*** | -0.29*** |
|  | 0.25, 0.31 | 0.18, 0.23 | -0.31, -0.28 |
| **Voronoi Neighborhood Demographics** | 0.22*** | 0.20*** | -0.42*** |
|  | 0.20, 0.24 | 0.18, 0.23 | -0.44, -0.41 |
| **Voronoi Neighborhood Poverty** | 0.59*** | 0.57*** | 0.13*** |
|  | 0.56, 0.62 | 0.53, 0.60 | 0.10, 0.17 |
| **Constant** | 27.38*** | 27.36*** | 31.85*** |
|  | 26.09, 28.67 | 26.08, 28.64 | 30.62, 33.07 |
| **Observations** | 22,704 | 22,704 | 22,704 |
| **R2** | 0.92 | 0.92 | 0.92 |
| **High Schools** | | | |
|  | **Black** | **Hispanic** | **White** |
| **School Demographic Residual** | 0.35*** | 0.28*** | -0.29*** |
|  | 0.31, 0.39 | 0.23, 0.33 | -0.32, -0.26 |
| **Voronoi Neighborhood Demographics** | 0.30*** | 0.28*** | -0.46*** |
|  | 0.26, 0.33 | 0.23, 0.33 | -0.48, -0.43 |
| **Voronoi Neighborhood Poverty** | 0.54*** | 0.56*** | 0.11*** |
|  | 0.48, 0.60 | 0.51, 0.62 | 0.05, 0.17 |
| **Constant** | 27.31*** | 27.22*** | 32.00*** |
|  | 25.85, 28.78 | 25.75, 28.69 | 30.58, 33.42 |
| **Observations** | 10,469 | 10,469 | 10,469 |
| **R2** | 0.90 | 0.90 | 0.91 |

| **S17: OLS Results, student/population race and ethnicity differences in September afternoon school LST for year 2016 weather and demographics, school catchment as the neighborhood, state as the region, and poverty as the neighborhood SES measure** | | | |
| --- | --- | --- | --- |
| **Elementary Schools** | | | |
|  | **Black** | **Hispanic** | **White** |
| **School Demographic Residuals** | 0.30*** | 0.20*** | -0.31*** |
|  | 0.26, 0.33 | 0.17, 0.22 | -0.33, -0.29 |
| **Catchment Demographics** | 0.25*** | 0.46*** | -0.48*** |
|  | 0.24, 0.27 | 0.44, 0.47 | -0.50, -0.47 |
| **Catchment Poverty** | 0.55*** | 0.40*** | 0.05** |
|  | 0.52, 0.58 | 0.37, 0.43 | 0.02, 0.08 |
| **Constant** | 30.06*** | 30.94*** | 34.97*** |
|  | 29.84, 30.28 | 30.73, 31.15 | 34.73, 35.20 |
| **Observations** | 36,724 | 36,724 | 36,724 |
| **R2** | 0.81 | 0.82 | 0.83 |
| **Middle Schools** | | | |
|  | **Black** | **Hispanic** | **White** |
| **School Demographic Residuals** | 0.33*** | 0.23*** | -0.29*** |
|  | 0.29, 0.38 | 0.19, 0.27 | -0.32, -0.26 |
| **Catchment Demographics** | 0.31*** | 0.49*** | -0.52*** |
|  | 0.28, 0.33 | 0.47, 0.51 | -0.54, -0.51 |
| **Catchment Poverty** | 0.54*** | 0.39*** | 0.00 |
|  | 0.50, 0.58 | 0.35, 0.43 | -0.04, 0.05 |
| **Constant** | 29.75*** | 30.59*** | 35.18*** |
|  | 29.47, 30.02 | 30.32, 30.85 | 34.87, 35.49 |
| **Observations** | 22,704 | 22,704 | 22,704 |
| **R2** | 0.82 | 0.83 | 0.84 |
| **High Schools** | | | |
|  | **Black** | **Hispanic** | **White** |
| **School Demographic Residuals** | 0.44*** | 0.29*** | -0.27*** |
|  | 0.37, 0.51 | 0.21, 0.36 | -0.32, -0.22 |
| **Catchment Demographics** | 0.34*** | 0.55*** | -0.51*** |
|  | 0.30, 0.38 | 0.51, 0.59 | -0.54, -0.49 |
| **Catchment Poverty** | 0.45*** | 0.30*** | -0.03 |
|  | 0.39, 0.52 | 0.24, 0.37 | -0.10, 0.04 |
| **Constant** | 29.81*** | 30.70*** | 35.12*** |
|  | 29.45, 30.16 | 30.35, 31.04 | 34.69, 35.55 |
| **Observations** | 10,469 | 10,469 | 10,469 |
| **R2** | 0.79 | 0.80 | 0.81 |
|  |  |  |  |
|  |  |  |  |

| **S18: OLS Results, student/population race and ethnicity differences in September afternoon school LST for year 2016 weather and demographics, Voronoi neighborhood as the neighborhood, state as the region, and poverty as the neighborhood SES measure** | | | |
| --- | --- | --- | --- |
| **Elementary Schools** | | | |
|  | **Black** | **Hispanic** | **White** |
| **School Demographic Residuals** | 0.28*** | 0.19*** | -0.29*** |
|  | 0.25, 0.30 | 0.17, 0.22 | -0.31, -0.27 |
| **Voronoi Neighborhood Demographics** | 0.26*** | 0.47*** | -0.49*** |
|  | 0.24, 0.27 | 0.46, 0.49 | -0.50, -0.47 |
| **Voronoi Neighborhood Poverty** | 0.57*** | 0.41*** | 0.07*** |
|  | 0.54, 0.60 | 0.38, 0.44 | 0.04, 0.10 |
| **Constant** | 30.05*** | 30.94*** | 35.00*** |
|  | 29.83, 30.27 | 30.72, 31.15 | 34.76, 35.24 |
| **Observations** | 36,724 | 36,724 | 36,724 |
| **R2** | 0.81 | 0.82 | 0.83 |
| **Middle Schools** | | | |
|  | **Black** | **Hispanic** | **White** |
| **School Demographic Residuals** | 0.33*** | 0.23*** | -0.29*** |
|  | 0.29, 0.37 | 0.20, 0.27 | -0.31, -0.26 |
| **Voronoi Neighborhood Demographics** | 0.30*** | 0.50*** | -0.52*** |
|  | 0.28, 0.32 | 0.48, 0.53 | -0.54, -0.50 |
| **Voronoi Neighborhood Poverty** | 0.55*** | 0.39*** | 0.03 |
|  | 0.50, 0.59 | 0.35, 0.43 | -0.02, 0.07 |
| **Constant** | 29.77*** | 30.63*** | 35.17*** |
|  | 29.50, 30.04 | 30.37, 30.90 | 34.87, 35.48 |
| **Observations** | 22,704 | 22,704 | 22,704 |
| **R2** | 0.82 | 0.83 | 0.84 |
| **High Schools** | | | |
|  | **Black** | **Hispanic** | **White** |
| **School Demographic Residuals** | 0.36*** | 0.33*** | -0.26*** |
|  | 0.30, 0.42 | 0.27, 0.39 | -0.30, -0.22 |
| **Voronoi Neighborhood Demographics** | 0.35*** | 0.55*** | -0.51*** |
|  | 0.31, 0.39 | 0.52, 0.59 | -0.54, -0.49 |
| **Voronoi Neighborhood Poverty** | 0.42*** | 0.28*** | -0.03 |
|  | 0.35, 0.49 | 0.22, 0.35 | -0.10, 0.04 |
| **Constant** | 29.87*** | 30.80*** | 35.22*** |
|  | 29.51, 30.22 | 30.45, 31.14 | 34.80, 35.65 |
| **Observations** | 10,469 | 10,469 | 10,469 |
| **R2** | 0.79 | 0.8 | 0.81 |
|  |  |  |  |

| **S19: OLS Results, student/population race and ethnicity differences in September afternoon school LST for year 2016 weather and demographics, school catchment as the neighborhood, the 5-mile buffer as the region, and poverty as the neighborhood SES measure** | | | |
| --- | --- | --- | --- |
| **Elementary Schools** | | | |
|  | **Black** | **Hispanic** | **White** |
| **School Demographic Residuals** | 0.05*** | 0.06*** | -0.07*** |
|  | 0.03, 0.06 | 0.05, 0.07 | -0.07, -0.06 |
| **Catchment Demographics** | -0.02*** | 0.04*** | -0.02*** |
|  | -0.03, -0.01 | 0.03, 0.04 | -0.02, -0.01 |
| **Catchment Poverty** | 0.25*** | 0.22*** | 0.22*** |
|  | 0.24, 0.26 | 0.21, 0.23 | 0.21, 0.23 |
| **5-mile Average LST** | 0.96*** | 0.95*** | 0.96*** |
|  | 0.96, 0.97 | 0.95, 0.96 | 0.96, 0.96 |
| **Constant** | 1.50*** | 1.74*** | 1.76*** |
|  | 1.44, 1.56 | 1.68, 1.81 | 1.67, 1.86 |
| **Observations** | 36,724 | 36,724 | 36,724 |
| **R2** | 0.97 | 0.97 | 0.97 |
| **Middle Schools** | | | |
|  | **Black** | **Hispanic** | **White** |
| **School Demographic Residuals** | 0.06*** | 0.07*** | -0.06*** |
|  | 0.04, 0.08 | 0.05, 0.08 | -0.08, -0.05 |
| **Catchment Demographics** | -0.02*** | 0.04*** | -0.02*** |
|  | -0.02, -0.01 | 0.03, 0.05 | -0.03, -0.01 |
| **Catchment Poverty** | 0.23*** | 0.20*** | 0.20*** |
|  | 0.22, 0.25 | 0.19, 0.22 | 0.18, 0.21 |
| **5-mile Average LST** | 0.96*** | 0.96*** | 0.96*** |
|  | 0.96, 0.97 | 0.95, 0.96 | 0.96, 0.96 |
| **Constant** | 1.46*** | 1.73*** | 1.81*** |
|  | 1.40, 1.53 | 1.65, 1.81 | 1.69, 1.93 |
| **Observations** | 22,704 | 22,704 | 22,704 |
| **R2** | 0.97 | 0.97 | 0.97 |
| **High Schools** | | | |
|  | **Black** | **Hispanic** | **White** |
| **School Demographic Residuals** | 0.08*** | 0.07*** | -0.06*** |
|  | 0.05, 0.10 | 0.04, 0.10 | -0.08, -0.04 |
| **Catchment Demographics** | -0.01 | 0.02** | -0.01 |
|  | -0.02, 00.00 | 0.01, 0.03 | -0.02, 00.00 |
| **Catchment Poverty** | 0.25*** | 0.24*** | 0.24*** |
|  | 0.23, 0.28 | 0.22, 0.27 | 0.21, 0.26 |
| **5-mile Average LST** | 0.96*** | 0.96*** | 0.96*** |
|  | 0.96, 0.97 | 0.95, 0.96 | 0.96, 0.96 |
| **Constant** | 1.51*** | 1.65*** | 1.66*** |
|  | 1.41, 1.62 | 1.53, 1.78 | 1.47, 1.86 |
| **Observations** | 10,469 | 10,469 | 10,469 |
| **R2** | 0.97 | 0.97 | 0.97 |
|  |  |  |  |
|  |  |  |  |

| **S20: OLS Results, student/population race and ethnicity differences in September afternoon school LST for year 2016 weather and demographics, school catchment as the neighborhood, the 10-mile buffer as the region, and poverty as the neighborhood SES measure** | | | |
| --- | --- | --- | --- |
| **Elementary Schools** | | | |
|  | **Black** | **Hispanic** | **White** |
| **School Demographic Residuals** | 0.12*** | 0.13*** | -0.16*** |
|  | 0.10, 0.13 | 0.11, 0.14 | -0.17, -0.14 |
| **Catchment Demographics** | 0.01** | 0.08*** | -0.08*** |
|  | 00.00, 0.02 | 0.07, 0.08 | -0.09, -0.08 |
| **Catchment Poverty** | 0.41*** | 0.39*** | 0.32*** |
|  | 0.40, 0.43 | 0.37, 0.40 | 0.30, 0.33 |
| **10-mile Average LST** | 0.95*** | 0.93*** | 0.95*** |
|  | 0.95, 0.95 | 0.93, 0.93 | 0.95, 0.95 |
| **Constant** | 2.11*** | 2.66*** | 3.47*** |
|  | 2.04, 2.19 | 2.57, 2.75 | 3.34, 3.60 |
| **Observations** | 36,724 | 36,724 | 36,724 |
| **R2** | 0.94 | 0.94 | 0.94 |
| **Middle Schools** | | | |
|  | **Black** | **Hispanic** | **White** |
| **School Demographic Residuals** | 0.12*** | 0.15*** | -0.15*** |
|  | 0.09, 0.14 | 0.13, 0.17 | -0.17, -0.13 |
| **Catchment Demographics** | 0.02** | 0.09*** | -0.10*** |
|  | 0.01, 0.03 | 0.08, 0.10 | -0.11, -0.09 |
| **Catchment Poverty** | 0.40*** | 0.37*** | 0.29*** |
|  | 0.38, 0.42 | 0.35, 0.39 | 0.26, 0.31 |
| **10-mile Average LST** | 0.95*** | 0.93*** | 0.93*** |
|  | 0.95, 0.95 | 0.92, 0.93 | 0.92, 0.93 |
| **Constant** | 2.04*** | 2.67*** | 3.61*** |
|  | 1.94, 2.13 | 2.56, 2.78 | 3.45, 3.77 |
| **Observations** | 22,704 | 22,704 | 22,704 |
| **R2** | 0.95 | 0.95 | 0.95 |
| **High Schools** | | | |
|  | **Black** | **Hispanic** | **White** |
| **School Demographic Residuals** | 0.16*** | 0.17*** | -0.16*** |
|  | 0.13, 0.20 | 0.13, 0.21 | -0.18, -0.13 |
| **Catchment Demographics** | 0.03** | 0.06*** | -0.08*** |
|  | 0.01, 0.04 | 0.04, 0.08 | -0.09, -0.06 |
| **Catchment Poverty** | 0.41*** | 0.42*** | 0.33*** |
|  | 0.38, 0.45 | 0.38, 0.45 | 0.30, 0.37 |
| **10-mile Average LST** | 0.95*** | 0.93*** | 0.93*** |
|  | 0.94, 0.95 | 0.93, 0.94 | 0.92, 0.93 |
| **Constant** | 2.06*** | 2.48*** | 3.31*** |
|  | 1.92, 2.21 | 2.31, 2.65 | 3.04, 3.57 |
| **Observations** | 10,469 | 10,469 | 10,469 |
| **R2** | 0.94 | 0.94 | 0.94 |
|  |  |  |  |
|  |  |  |  |

| **S21: OLS Results, student/population race and ethnicity differences in September afternoon school LST for year 2016 weather and demographics, school catchment as the neighborhood, the 25-mile buffer as the region, and poverty as the neighborhood SES measure** | | | |
| --- | --- | --- | --- |
| **Elementary Schools** | | | |
|  | **Black** | **Hispanic** | **White** |
| **School Demographic Residuals** | 0.21*** | 0.20*** | -0.26*** |
|  | 0.19, 0.23 | 0.18, 0.22 | -0.28, -0.25 |
| **Catchment Demographics** | 0.11*** | 0.15*** | -0.21*** |
|  | 0.10, 0.12 | 0.14, 0.16 | -0.22, -0.20 |
| **Catchment Poverty** | 0.50*** | 0.51*** | 0.31*** |
|  | 0.48, 0.53 | 0.49, 0.53 | 0.29, 0.33 |
| **25-mile Average LST** | 0.94*** | 0.90*** | 0.88*** |
|  | 0.93, 0.94 | 0.89, 0.90 | 0.88, 0.89 |
| **Constant** | 2.85*** | 3.97*** | 6.23*** |
|  | 2.75, 2.95 | 3.85, 4.08 | 6.07, 6.38 |
| **Observations** | 36,724 | 36,724 | 36,724 |
| **R2** | 0.90 | 0.91 | 0.91 |
| **Middle Schools** | | | |
|  | **Black** | **Hispanic** | **White** |
| **School Demographic Residuals** | 0.19*** | 0.25*** | -0.26*** |
|  | 0.16, 0.23 | 0.22, 0.28 | -0.28, -0.24 |
| **Catchment Demographics** | 0.13*** | 0.18*** | -0.24*** |
|  | 0.12, 0.15 | 0.16, 0.19 | -0.25, -0.23 |
| **Catchment Poverty** | 0.49*** | 0.50*** | 0.26*** |
|  | 0.46, 0.52 | 0.47, 0.52 | 0.23, 0.29 |
| **25-mile Average LST** | 0.94*** | 0.89*** | 0.88*** |
|  | 0.94, 0.94 | 0.89, 0.90 | 0.88, 0.89 |
| **Constant** | 2.69*** | 3.93*** | 6.50*** |
|  | 2.57, 2.81 | 3.79, 4.08 | 6.30, 6.70 |
| **Observations** | 22,704 | 22,704 | 22,704 |
| **R2** | 0.91 | 0.91 | 0.92 |
| **High Schools** | | | |
|  | **Black** | **Hispanic** | **White** |
| **School Demographic Residuals** | 0.32*** | 0.27*** | -0.27*** |
|  | 0.27, 0.37 | 0.22, 0.32 | -0.31, -0.24 |
| **Catchment Demographics** | 0.12*** | 0.15*** | -0.21*** |
|  | 0.10, 0.15 | 0.13, 0.17 | -0.23, -0.19 |
| **Catchment Poverty** | 0.51*** | 0.54*** | 0.32*** |
|  | 0.47, 0.56 | 0.50, 0.59 | 0.28, 0.37 |
| **25-mile Average LST** | 0.94*** | 0.90*** | 0.89*** |
|  | 0.93, 0.94 | 0.89, 0.91 | 0.88, 0.90 |
| **Constant** | 2.61*** | 3.58*** | 5.92*** |
|  | 2.43, 2.80 | 3.35, 3.80 | 5.60, 6.25 |
| **Observations** | 10,469 | 10,469 | 10,469 |
| **R2** | 0.90 | 0.90 | 0.90 |
|  |  |  |  |
|  |  |  |  |

| **S22: OLS Results, student/population race and ethnicity differences in September afternoon school LST for year 2016 weather and demographics, the Voronoi neighborhood as the neighborhood, the 5-mile buffer as the region, and poverty as the neighborhood SES measure** | | | |
| --- | --- | --- | --- |
| **Elementary Schools** | | | |
|  | **Black** | **Hispanic** | **White** |
| **School Demographic Residuals** | 0.03*** | 0.05*** | -0.05*** |
|  | 0.02, 0.04 | 0.04, 0.06 | -0.06, -0.04 |
| **Voronoi Neighborhood Demographics** | -0.02*** | 0.04*** | -0.02*** |
|  | -0.03, -0.01 | 0.03, 0.05 | -0.02, -0.01 |
| **Voronoi Neighborhood Poverty** | 0.26*** | 0.23*** | 0.23*** |
|  | 0.25, 0.27 | 0.22, 0.24 | 0.21, 0.24 |
| **5-mile Average LST** | 0.96*** | 0.95*** | 0.96*** |
|  | 0.96, 0.96 | 0.95, 0.96 | 0.96, 0.96 |
| **Constant** | 1.50*** | 1.76*** | 1.81*** |
|  | 1.44, 1.56 | 1.69, 1.82 | 1.71, 1.91 |
| **Observations** | 36,724 | 36,724 | 36,724 |
| **R2** | 0.97 | 0.97 | 0.97 |
| **Middle Schools** |  |  |  |
|  | **Black** | **Hispanic** | **White** |
| **School Demographic Residuals** | 0.04*** | 0.05*** | -0.05*** |
|  | 0.03, 0.06 | 0.04, 0.06 | -0.06, -0.04 |
| **Voronoi Neighborhood Demographics** | -0.02*** | 0.04*** | -0.02*** |
|  | -0.02, -0.01 | 0.04, 0.05 | -0.03, -0.02 |
| **Voronoi Neighborhood Poverty** | 0.24*** | 0.21*** | 0.20*** |
|  | 0.23, 0.26 | 0.20, 0.23 | 0.19, 0.22 |
| **5-mile Average LST** | 0.96*** | 0.95*** | 0.96*** |
|  | 0.96, 0.97 | 0.95, 0.96 | 0.96, 0.96 |
| **Constant** | 1.46*** | 1.74*** | 1.85*** |
|  | 1.40, 1.53 | 1.66, 1.82 | 1.73, 1.97 |
| **Observations** | 22,704 | 22,704 | 22,704 |
| **R2** | 0.97 | 0.97 | 0.97 |
| **High Schools** | | | |
|  | **Black** | **Hispanic** | **White** |
| **School Demographic Residuals** | 0.05*** | 0.05*** | -0.04*** |
|  | 0.03, 0.07 | 0.03, 0.07 | -0.06, -0.03 |
| **Voronoi Neighborhood Demographics** | -0.01 | 0.02*** | -0.01** |
|  | -0.02, 0.01 | 0.01, 0.04 | -0.02, -00.00 |
| **Voronoi Neighborhood Poverty** | 0.26*** | 0.25*** | 0.24*** |
|  | 0.23, 0.28 | 0.22, 0.27 | 0.21, 0.26 |
| **5-mile Average LST** | 0.96*** | 0.96*** | 0.96*** |
|  | 0.96, 0.97 | 0.95, 0.96 | 0.95, 0.96 |
| **Constant** | 1.51*** | 1.68*** | 1.77*** |
|  | 1.41, 1.62 | 1.55, 1.80 | 1.57, 1.96 |
| **Observations** | 10,469 | 10,469 | 10,469 |
| **R2** | 0.97 | 0.97 | 0.97 |
|  |  |  |  |
|  |  |  |  |

| **S23: OLS Results, student/population race and ethnicity differences in September afternoon school LST for year 2016 weather and demographics, the Voronoi neighborhood as the neighborhood, the 10-mile buffer as the region, and poverty as the neighborhood SES measure** | | | |
| --- | --- | --- | --- |
| **Elementary Schools** | | | |
|  | **Black** | **Hispanic** | **White** |
| **School Demographic Residuals** | 0.09*** | 0.11*** | -0.13*** |
|  | 0.08, 0.11 | 0.10, 0.13 | -0.14, -0.12 |
| **Voronoi Neighborhood Demographics** | 0.01** | 0.08*** | -0.09*** |
|  | 0.00, 0.02 | 0.07, 0.09 | -0.09, -0.08 |
| **Voronoi Neighborhood Poverty** | 0.43*** | 0.40*** | 0.33*** |
|  | 0.41, 0.45 | 0.39, 0.42 | 0.32, 0.35 |
| **10-mile Average LST** | 0.95*** | 0.93*** | 0.93*** |
|  | 0.95, 0.95 | 0.92, 0.93 | 0.92, 0.93 |
| **Constant** | 2.12*** | 2.68*** | 3.54*** |
|  | 2.04, 2.19 | 2.59, 2.77 | 3.41, 3.67 |
| **Observations** | 36,724 | 36,724 | 36,724 |
| **R2** | 0.94 | 0.94 | 0.94 |
| **Middle Schools** | | | |
|  | **Black** | **Hispanic** | **White** |
| **School Demographic Residuals** | 0.10*** | 0.13*** | -0.12*** |
|  | 0.08, 0.12 | 0.11, 0.15 | -0.14, -0.11 |
| **Voronoi Neighborhood Demographics** | 0.02** | 0.10*** | -0.10*** |
|  | 0.01, 0.03 | 0.09, 0.11 | -0.11, -0.09 |
| **Voronoi Neighborhood Poverty** | 0.41*** | 0.38*** | 0.30*** |
|  | 0.39, 0.43 | 0.36, 0.40 | 0.28, 0.32 |
| **10-mile Average LST** | 0.95*** | 0.93*** | 0.93*** |
|  | 0.95, 0.95 | 0.92, 0.93 | 0.92, 0.93 |
| **Constant** | 2.04*** | 2.70*** | 3.68*** |
|  | 1.94, 2.13 | 2.58, 2.81 | 3.51, 3.84 |
| **Observations** | 22,704 | 22,704 | 22,704 |
| **R2** | 0.95 | 0.95 | 0.95 |
| **High Schools** | | | |
|  | **Black** | **Hispanic** | **White** |
| **School Demographic Residuals** | 0.12*** | 0.14*** | -0.12*** |
|  | 0.09, 0.15 | 0.11, 0.17 | -0.14, -0.10 |
| **Voronoi Neighborhood Demographics** | 0.03*** | 0.07*** | -0.08*** |
|  | 0.01, 0.05 | 0.05, 0.08 | -0.10, -0.07 |
| **Voronoi Neighborhood Poverty** | 0.40*** | 0.41*** | 0.33*** |
|  | 0.37, 0.44 | 0.37, 0.44 | 0.30, 0.37 |
| **10-mile Average LST** | 0.95*** | 0.93*** | 0.93*** |
|  | 0.94, 0.95 | 0.93, 0.94 | 0.92, 0.93 |
| **Constant** | 2.08*** | 2.53*** | 3.46*** |
|  | 1.93, 2.22 | 2.36, 2.71 | 3.20, 3.72 |
| **Observations** | 10,469 | 10,469 | 10,469 |
| **R2** | 0.94 | 0.94 | 0.94 |
|  |  |  |  |
|  |  |  |  |

| **S24: OLS Results, student/population race and ethnicity differences in September afternoon school LST for year 2016 weather and demographics, the Voronoi neighborhood as the neighborhood, the 25-mile buffer as the region, and poverty as the neighborhood SES measure** | | | |
| --- | --- | --- | --- |
| **Elementary Schools** | | | |
|  | **Black** | **Hispanic** | **White** |
| **School Demographic Residuals** | 0.18*** | 0.18*** | -0.22*** |
|  | 0.16, 0.20 | 0.16, 0.19 | -0.24, -0.21 |
| **Voronoi Neighborhood Demographics** | 0.11*** | 0.15*** | -0.22*** |
|  | 0.10, 0.13 | 0.14, 0.17 | -0.23, -0.21 |
| **Voronoi Neighborhood Poverty** | 0.52*** | 0.53*** | 0.33*** |
|  | 0.50, 0.54 | 0.51, 0.55 | 0.31, 0.35 |
| **25-mile Average LST** | 0.94*** | 0.89*** | 0.88*** |
|  | 0.93, 0.94 | 0.89, 0.90 | 0.88, 0.89 |
| **Constant** | 2.86*** | 4.01*** | 6.33*** |
|  | 2.76, 2.96 | 3.89, 4.12 | 6.17, 6.49 |
| **Observations** | 36,724 | 36,724 | 36,724 |
| **R2** | 0.9 | 0.91 | 0.91 |
| **Middle Schools** | | | |
|  | **Black** | **Hispanic** | **White** |
| **School Demographic Residuals** | 0.18*** | 0.22*** | -0.22*** |
|  | 0.15, 0.20 | 0.19, 0.24 | -0.24, -0.21 |
| **Voronoi Neighborhood Demographics** | 0.13*** | 0.18*** | -0.24*** |
|  | 0.12, 0.15 | 0.17, 0.20 | -0.25, -0.23 |
| **Voronoi Neighborhood Poverty** | 0.50*** | 0.50*** | 0.28*** |
|  | 0.47, 0.53 | 0.48, 0.53 | 0.25, 0.31 |
| **25-mile Average LST** | 0.94*** | 0.89*** | 0.88*** |
|  | 0.94, 0.94 | 0.89, 0.90 | 0.88, 0.89 |
| **Constant** | 2.69*** | 3.98*** | 6.55*** |
|  | 2.57, 2.81 | 3.84, 4.12 | 6.35, 6.75 |
| **Observations** | 22,704 | 22,704 | 22,704 |
| **R2** | 0.91 | 0.91 | 0.92 |
| **High Schools** | | | |
|  | **Black** | **Hispanic** | **White** |
| **School Demographic Residuals** | 0.24*** | 0.25*** | -0.23*** |
|  | 0.20, 0.27 | 0.21, 0.29 | -0.25, -0.20 |
| **Voronoi Neighborhood Demographics** | 0.13*** | 0.15*** | -0.22*** |
|  | 0.11, 0.16 | 0.13, 0.18 | -0.23, -0.20 |
| **Voronoi Neighborhood Poverty** | 0.48*** | 0.52*** | 0.32*** |
|  | 0.44, 0.53 | 0.47, 0.56 | 0.27, 0.36 |
| **25-mile Average LST** | 0.94*** | 0.90*** | 0.89*** |
|  | 0.93, 0.94 | 0.89, 0.91 | 0.88, 0.89 |
| **Constant** | 2.65*** | 3.67*** | 6.12*** |
|  | 2.47, 2.84 | 3.45, 3.90 | 5.79, 6.44 |
| **Observations** | 10,469 | 10,469 | 10,469 |
| **R2** | 0.90 | 0.90 | 0.90 |
|  |  |  |  |
|  |  |  |  |

| **S25: OLS Results, student/population race and ethnicity differences in September afternoon school LST for year 2016 weather and demographics, school catchment as the neighborhood, MSA as the region, and median household income as the neighborhood SES measure** | | | |
| --- | --- | --- | --- |
| **Elementary Schools** | | | |
|  | **Black** | **Hispanic** | **White** |
| **School Demographic Residuals** | 0.32*** | 0.19*** | -0.35*** |
|  | 0.29, 0.34 | 0.16, 0.21 | -0.37, -0.34 |
| **Catchment Demographics** | 0.27*** | 0.29*** | -0.40*** |
|  | 0.25, 0.28 | 0.27, 0.31 | -0.41, -0.39 |
| **Catchment Median Household Income** | -0.17*** | -0.15*** | -0.04*** |
|  | -0.18, -0.16 | -0.16, -0.14 | -0.05, -0.04 |
| **Constant** | 29.48*** | 29.31*** | 32.60*** |
|  | 28.34, 30.63 | 28.17, 30.45 | 31.53, 33.68 |
| **Observations** | 32,601 | 32,601 | 32,601 |
| **R2** | 0.91 | 0.91 | 0.92 |
| **Middle Schools** | | | |
|  | **Black** | **Hispanic** | **White** |
| **School Demographic Residuals** | 0.31*** | 0.22*** | -0.33*** |
|  | 0.27, 0.35 | 0.18, 0.25 | -0.36, -0.31 |
| **Catchment Demographics** | 0.28*** | 0.31*** | -0.42*** |
|  | 0.26, 0.31 | 0.28, 0.33 | -0.43, -0.40 |
| **Catchment Median Household Income** | -0.17*** | -0.13*** | -0.03*** |
|  | -0.18, -0.15 | -0.15, -0.12 | -0.04, -0.02 |
| **Constant** | 28.82*** | 28.59*** | 32.08*** |
|  | 27.54, 30.10 | 27.31, 29.87 | 30.87, 33.29 |
| **Observations** | 19,079 | 19,079 | 19,079 |
| **R2** | 0.92 | 0.92 | 0.93 |
| **High Schools** | | | |
|  | **Black** | **Hispanic** | **White** |
| **School Demographic Residuals** | 0.48*** | 0.34*** | -0.33*** |
|  | 0.40, 0.55 | 0.26, 0.42 | -0.37, -0.28 |
| **Catchment Demographics** | 0.37*** | 0.42*** | -0.44*** |
|  | 0.32, 0.41 | 0.37, 0.47 | -0.47, -0.41 |
| **Catchment Median Household Income** | -0.08*** | -0.06*** | 0.01 |
|  | -0.10, -0.06 | -0.08, -0.03 | -0.02, 0.03 |
| **Constant** | 28.18*** | 27.96*** | 31.84*** |
|  | 26.76, 29.60 | 26.54, 29.39 | 30.47, 33.22 |
| **Observations** | 8,006 | 8,006 | 8,006 |
| **R2** | 0.90 | 0.90 | 0.91 |

| **S26: OLS Results, student/population race and ethnicity differences in September afternoon school LST for year 2016 weather and demographics, Voronoi neighborhood as the neighborhood, MSA as the region, and median household income as the neighborhood SES measure** | | | |
| --- | --- | --- | --- |
| **Elementary Schools** | | | |
|  | **Black** | **Hispanic** | **White** |
| **School Demographic Residuals** | 0.28*** | 0.17*** | -0.30*** |
|  | 0.25, 0.30 | 0.15, 0.19 | -0.31, -0.28 |
| **Voronoi Neighborhood Demographics** | 0.27*** | 0.31*** | -0.40*** |
|  | 0.25, 0.28 | 0.29, 0.32 | -0.41, -0.39 |
| **Voronoi Neighborhood Median Household Income** | -0.18*** | -0.15*** | -0.06*** |
|  | -0.19, -0.17 | -0.16, -0.14 | -0.07, -0.05 |
| **Constant** | 29.58*** | 29.39*** | 32.74*** |
|  | 28.44, 30.72 | 28.25, 30.53 | 31.67, 33.81 |
| **Observations** | 32,978 | 32,978 | 32,978 |
| **R2** | 0.91 | 0.91 | 0.92 |
| **Middle Schools** | | | |
|  | **Black** | **Hispanic** | **White** |
| **School Demographic Residuals** | 0.31*** | 0.19*** | -0.29*** |
|  | 0.27, 0.34 | 0.16, 0.22 | -0.31, -0.27 |
| **Voronoi Neighborhood Demographics** | 0.27*** | 0.32*** | -0.41*** |
|  | 0.25, 0.29 | 0.29, 0.34 | -0.43, -0.40 |
| **Voronoi Neighborhood Median Household Income** | -0.18*** | -0.15*** | -0.06*** |
|  | -0.20, -0.17 | -0.16, -0.14 | -0.07, -0.04 |
| **Constant** | 28.94*** | 28.69*** | 32.20*** |
|  | 27.67, 30.22 | 27.41, 29.96 | 30.99, 33.40 |
| **Observations** | 19,987 | 19,987 | 19,987 |
| **R2** | 0.92 | 0.92 | 0.93 |
| **High Schools** | | | |
|  | **Black** | **Hispanic** | **White** |
| **School Demographic Residuals** | 0.37*** | 0.29*** | -0.29*** |
|  | 0.32, 0.42 | 0.23, 0.34 | -0.32, -0.26 |
| **Voronoi Neighborhood Demographics** | 0.37*** | 0.42*** | -0.46*** |
|  | 0.33, 0.41 | 0.38, 0.46 | -0.48, -0.43 |
| **Voronoi Neighborhood Median Household Income** | -0.10*** | -0.08*** | -0.01 |
|  | -0.12, -0.08 | -0.10, -0.06 | -0.03, 0.01 |
| **Constant** | 28.43*** | 28.17*** | 32.18*** |
|  | 26.98, 29.88 | 26.72, 29.62 | 30.80, 33.56 |
| **Observations** | 8,873 | 8,873 | 8,873 |
| **R2** | 0.91 | 0.90 | 0.92 |
|  |  |  |  |

| **S27: OLS Results, student/population race and ethnicity differences in September afternoon school LST for year 2016 weather and demographics, school catchment as the neighborhood, MSA as the region, and median home value as the neighborhood SES measure** | | | |
| --- | --- | --- | --- |
| **Elementary Schools** | | | |
|  | **Black** | **Hispanic** | **White** |
| **School Demographic Residuals** | 0.37*** | 0.22*** | -0.37*** |
|  | 0.34, 0.40 | 0.20, 0.25 | -0.39, -0.35 |
| **Catchment Demographics** | 0.30*** | 0.32*** | -0.38*** |
|  | 0.28, 0.31 | 0.31, 0.34 | -0.39, -0.37 |
| **Catchment Median Home Value** | -0.03*** | -0.03*** | -0.02*** |
|  | -0.04, -0.03 | -0.03, -0.03 | -0.02, -0.01 |
| **Constant** | 28.85*** | 28.73*** | 32.38*** |
|  | 27.64, 30.05 | 27.52, 29.94 | 31.25, 33.52 |
| **Observations** | 30,375 | 30,375 | 30,375 |
| **R2** | 0.90 | 0.90 | 0.91 |
| **Middle Schools** | | | |
|  | **Black** | **Hispanic** | **White** |
| **School Demographic Residuals** | 0.35*** | 0.24*** | -0.33*** |
|  | 0.31, 0.40 | 0.20, 0.27 | -0.36, -0.30 |
| **Catchment Demographics** | 0.31*** | 0.33*** | -0.40*** |
|  | 0.29, 0.33 | 0.30, 0.35 | -0.42, -0.38 |
| **Catchment Median Home Value** | -0.03*** | -0.03*** | -0.02*** |
|  | -0.04, -0.03 | -0.03, -0.02 | -0.02, -0.01 |
| **Constant** | 28.02*** | 27.90*** | 31.74*** |
|  | 26.67, 29.38 | 26.54, 29.26 | 30.45, 33.03 |
| **Observations** | 17,273 | 17,273 | 17,273 |
| **R2** | 0.92 | 0.93 | 0.89 |
| **High Schools** | | | |
|  | **Black** | **Hispanic** | **White** |
| **School Demographic Residuals** | 0.46*** | 0.31*** | -0.26*** |
|  | 0.37, 0.55 | 0.22, 0.39 | -0.31, -0.20 |
| **Catchment Demographics** | 0.36*** | 0.43*** | -0.43*** |
|  | 0.31, 0.41 | 0.38, 0.49 | -0.46, -0.40 |
| **Catchment Median Home Value** | -0.02*** | -0.02*** | -0.01*** |
|  | -0.03, -0.02 | -0.02, -0.01 | -0.02, -0.01 |
| **Constant** | 27.85*** | 27.70*** | 31.88*** |
|  | 26.32, 29.38 | 26.16, 29.23 | 30.38, 33.38 |
| **Observations** | 7,156 | 7,156 | 7,156 |
| **R2** | 0.89 | 0.89 | 0.90 |

| **S28: OLS Results, student/population race and ethnicity differences in September afternoon school LST for year 2016 weather and demographics, Voronoi neighborhood as the neighborhood, MSA as the region, and median home value as the neighborhood SES measure** | | | |
| --- | --- | --- | --- |
| **Elementary Schools** | | | |
|  | **Black** | **Hispanic** | **White** |
| **School Demographic Residuals** | 0.31*** | 0.19*** | -0.31*** |
|  | 0.29, 0.34 | 0.17, 0.21 | -0.33, -0.29 |
| **Voronoi Neighborhood Demographics** | 0.30*** | 0.33*** | -0.39*** |
|  | 0.28, 0.31 | 0.31, 0.35 | -0.40, -0.38 |
| **Voronoi Neighborhood Median Home Value** | -0.04*** | -0.03*** | -0.02*** |
|  | -0.04, -0.03 | -0.03, -0.03 | -0.02, -0.02 |
| **Constant** | 28.90*** | 28.77*** | 32.46*** |
|  | 27.71, 30.10 | 27.57, 29.97 | 31.33, 33.59 |
| **Observations** | 30,710 | 30,710 | 30,710 |
| **R2** | 0.90 | 0.90 | 0.92 |
| **Middle Schools** | | | |
|  | **Black** | **Hispanic** | **White** |
| **School Demographic Residuals** | 0.33*** | 0.22*** | -0.30*** |
|  | 0.29, 0.36 | 0.19, 0.25 | -0.33, -0.28 |
| **Voronoi Neighborhood Demographics** | 0.30*** | 0.33*** | -0.40*** |
|  | 0.28, 0.32 | 0.31, 0.35 | -0.41, -0.38 |
| **Voronoi Neighborhood Median Home Value** | -0.04*** | -0.03*** | -0.02*** |
|  | -0.04, -0.03 | -0.03, -0.03 | -0.02, -0.02 |
| **Constant** | 28.27*** | 28.13*** | 31.95*** |
|  | 27.01, 29.53 | 26.87, 29.40 | 30.75, 33.15 |
| **Observations** | 18,551 | 18,551 | 18,551 |
| **R2** | 0.92 | 0.92 | 0.93 |
| **High Schools** | | | |
|  | **Black** | **Hispanic** | **White** |
| **School Demographic Residuals** | 0.39*** | 0.29*** | -0.30*** |
|  | 0.34, 0.44 | 0.24, 0.35 | -0.33, -0.26 |
| **Voronoi Neighborhood Demographics** | 0.37*** | 0.41*** | -0.44*** |
|  | 0.33, 0.41 | 0.37, 0.46 | -0.46, -0.41 |
| **Voronoi Neighborhood Median Home Value** | -0.03*** | -0.02*** | -0.01*** |
|  | -0.03, -0.02 | -0.03, -0.02 | -0.02, -0.01 |
| **Constant** | 28.10*** | 27.94*** | 32.11*** |
|  | 26.67, 29.52 | 26.50, 29.37 | 30.73, 33.49 |
| **Observations** | 8,274 | 8,274 | 8,274 |
| **R2** | 0.90 | 0.90 | 0.91 |
